# Associations of Gestational Exposure to Fine Particulate Matter Constituents with Preterm Birth: A Birth Cohort-Based Hypothetical Intervention Study

**DOI:** 10.3390/toxics14030233

**Published:** 2026-03-09

**Authors:** Yonggui Gao, Rui Qian, Xinyue Li, Sheng Qiu, Zijun Yang, Saijun Huang, Pengzhen Hu, Yin Yang, Hualiang Lin, Xi Su, Qingmei Lin, Zilong Zhang

**Affiliations:** 1 Department of Epidemiology, School of Public Health, Sun Yat-sen University, Guangzhou 510080, China; gaoyg5@mail2.sysu.edu.cn (Y.G.); lixy856@mail2.sysu.edu.cn (X.L.); yangzj39@mail2.sysu.edu.cn (Z.Y.); yangyin3@mail.sysu.edu.cn (Y.Y.); linhualiang@mail.sysu.edu.cn (H.L.); 2 Statistical Information Center for Health and Family Planning of Foshan, Foshan 528000, China; charry811@163.com; 3 Nanhai Maternal and Children’s Hospital of Guangzhou University of Chinese Medicine, Foshan 528000, China; killer810803@163.com; 4 Foshan Women and Children Hospital Affiliated to Guangdong Medical University, Foshan 528315, China; tufanqie@163.com (S.H.); reynacuba2016@163.com (P.H.); fssuxi@sina.com (X.S.)

**Keywords:** fine particulate matter, chemical constituents, preterm birth, hypothetical intervention, g-computation

## Abstract

Preterm birth (PTB) has been increasingly linked to maternal exposure to fine particulate matter (PM_2.5_) during pregnancy. However, the contribution of individual PM_2.5_ constituents risk remains unclear. This research investigated associations between prenatal exposure to PM_2.5_ constituents and PTB risk using a hypothetical intervention approach. A birth cohort of 148,068 mother–child pairs from Foshan, China was constructed from health records. Maternal exposure to PM_2.5_ constituents—including black carbon (BC), organic matter (OM), nitrate (NO_3_**^−^**), ammonium (NH_4_^+^), and sulfate (SO_4_^2−^)—was estimated based on satellite-derived spatial and temporal modeling. Parametric G-computation and distributed lag nonlinear models were used to estimate the cumulative risks of PTB under hypothetical reductions of PM_2.5_ constituents during pregnancy. Potential benefits (preventable PTB cases) were also estimated. Among the cohort, 9757 (6.59%) PTBs were observed. Hypothetical reductions in all five constituents during pregnancy were associated with decreased cumulative risks of birth at week 36 (i.e., the threshold for PTB). For instance, a 40% reduction (reducing PM_2.5_ to the WHO recommended levels) yielded risk differences of −2.29 (BC), −3.70 (OM), −4.74 (NH_4_^+^), −5.00 (NO_3_**^−^**), and −2.11 (SO_4_^2−^) per thousand births, corresponding to 312 (3.20%) to 740 (7.58%) preventable cases. Our results indicate that reductions in PM_2.5_ constituents, especially NO_3_**^−^** and NH_4_^+^, were associated with lower risks of PTB.

## 1. Introduction

Globally, preterm birth (PTB) continues to be a major contributor to neonatal mortality [1]. Furthermore, PTB is also associated with increased risks of various childhood diseases (e.g., stunted growth, mental disability) [2,3] as well as adulthood cardiovascular disease, diabetes, and mental illness [4,5,6]. Global estimates suggest that PTB incidence reaches approximately 15 million annually, accounting for nearly 11% of live births [7]. Given the substantial disease burden caused by PTB, it is important to recognize the risk factors of PTB, which can inform the development of effective preventive measures.

The etiology of PTB involves intricate biological processes that are still only partially characterized. Multiple factors, including environmental exposures, genetic predisposition, and maternal health conditions, are recognized as potential contributors to PTB [8,9,10]. While numerous studies over the past decades have linked fine particulate matter (PM_2.5_) exposure to PTB [11,12,13], the associations of its constituents remain poorly understood. PM_2.5_ is a complex mixture mainly composed of inorganic elements, carbonaceous components, and organic compounds [14,15], and PM_2.5_ composition greatly varies both geographically and temporally. However, most previous studies only examined the health impact of PM_2.5_ total mass on PTB, neglecting the fact that PM_2.5_ with different constituents generally exhibit distinct toxic effects [16,17] and the involved biological pathways could also be different [18,19]. At the same time, PM_2.5_ constituents are closely related to the emission sources [20], and investigations on the specific PM_2.5_ constituents are therefore more relevant to the development of PM_2.5_ mitigation strategies from a policy perspective.

The associations between PM_2.5_ constituents and PTB have been explored in limited research, but conclusions remain inconsistent [21,22,23,24]. Notably, previous studies were mainly observational (e.g., case–control or cohort studies) which used traditional statistical models (e.g., logistic or Cox proportional hazards regressions), and their results were subject to confounding bias, especially residual confounding. In environmental epidemiology, the majority of research relies on observational designs since environmental exposures cannot typically be randomized. In other words, it is not feasible to conduct experimental epidemiological studies, especially randomized controlled trials, that can effectively deal with both measured and unmeasured confounding through randomization. However, recent advances in epidemiological methods provide alternative approaches such as the hypothetical interventions based on a counterfactual framework. In particular, the hypothetical intervention using the parametric G-computation can enhance confounding control and strengthen causal inference in observational studies by constructing the joint distribution of confounders, exposures, and outcomes in a parametric framework, thereby capturing their complex and dynamic interrelationships [25,26]. In recent years, this approach has been utilized in several observational studies into air pollution’s health effects, producing promising findings [27,28].

Based on a large birth cohort, we conducted a hypothetical intervention analysis using the parametric G-computation to evaluate the effect of reducing exposure to five major constituents of PM_2.5_ [black carbon (BC), organic matter (OM), nitrate (NO_3_**^−^**), ammonium (NH_4_^+^), and sulfate (SO_4_^2^**^−^**)] during pregnancy on the risk of PTB, which can facilitate the development of strategies for PM_2.5_ control and PTB prevention.

## 2. Materials and Methods

### 2.1. Study Population

Our study was based on a birth cohort in Foshan, Southern China, with more details available in our previous publications [29,30]. In brief, pregnant women registered at accredited healthcare institutions at their initial antenatal examinations were included and subsequently followed until delivery. Using unique identifiers, we linked mothers from the pregnancy registry to their offspring to construct the birth cohort. This study was approved by the Ethics Committee of School of Public Health, Sun Yat-sen University.

In the present study, we included 322,481 live births born between January 2019 and September 2023. Of these, only 299,442 singletons with gestations ≥ 28 weeks were considered. Participants with missing data on residential address or key covariates (*n* = 151,374) were excluded. Finally, a total of 148,068 mother–child pairs were included in the analysis (Figure S1). Overall, most general attributes—such as maternal age and infant sex—were similarly distributed in both the analytical and original cohorts. (Table S1).

### 2.2. Air Pollution Exposure Assessment

Air pollution exposure was estimated using datasets from the Tracking Air Pollution in China (TAP) platform (http://tapdata.org.cn, URL accessed from 1 February to 30 March 2025), which provides near-ground real-time air pollution data including PM_2.5_ across China from 2000 to 2024 at a 10 × 10 km resolution [31,32]. Briefly, TAP uses spatial-temporal models that were developed using machine learning algorithms and the integration of multi-source data, combining ground monitoring data, satellite remote sensing, meteorological reanalysis data, and emission inventories to estimate daily concentrations of PM_2.5_ and its five constituents, including BC, OM, NO_3_**^−^**, NH_4_^+^, and SO_4_^2^**^−^**. The TAP platform integrated data from 1640 and 571 ground monitoring stations for PM_2.5_ and its constituents, respectively, covering almost all provinces of China. The predicted concentrations of PM_2.5_ constituents showed decent correlations with real-time measurements from ground monitoring stations, with correlation coefficients ranging from 0.67 to 0.80 [32].

In our study, geocoding was used to translate pregnant women’s street-level residential addresses into longitude and latitude values. Subsequently, bilinear interpolation was used to assign the daily concentrations of air pollutants to each location. We computed week-specific concentrations of PM_2.5_ and its five constituents from the date of the last menstrual period (LMP) through to delivery.

### 2.3. Outcome Assessment

Using the date of conception, gestational age was estimated, and births occurring from 28 to 36 weeks were categorized as PTB, whereas those before 28 weeks were defined as miscarriages [33]. In this study, delivery was defined as a time-to-event survival outcome. The initiation date was set as the date of conception, and each gestational week was treated as a follow-up interval, during which time-varying exposures (air pollutants and temperature) were estimated. To estimate the risk of PTB, all pregnancies were classified as being at risk starting from the 28th week of gestation and were subsequently followed until one of the following events occurred: (a) delivery before 37 weeks of gestation (PTB); or (b) continuation of pregnancy until reaching 37 weeks of gestation, indicating an expected full-term delivery. Since the risk of PTB no longer existed beyond 37 weeks, all outcomes were treated as censored from the 37th week onward.

### 2.4. Covariates

We considered a group of covariates with reference to prior studies, including maternal age (years), maternal employment status (employed or unemployed), maternal pre-pregnancy body mass index (BMI, kg/m^2^), maternal educational attainment [categorized as high (college or above), medium (high school or equivalent technical school), or low (middle school or lower)], gestational diabetes mellitus (yes or no), gestational hypertension (yes or no), local household registration in Foshan (yes or no), and infant sex (male or female). We also included the year and season of conception as covariates to adjust for temporal trend and seasonal variations.

Previous studies have suggested that both nitrogen dioxide (NO_2_) exposure and unfavorable temperature during pregnancy are associated with increases in the risk of PTB [34,35]. To control for the possible impact of NO_2_ and temperature, we included them as time-varying covariates into the regression models. Daily average NO_2_ concentrations were sourced from the China High Air Pollutants dataset [36,37], while daily temperature data were obtained from the China Meteorological Administration Land Data Assimilation System [38]. For each participant, we calculated the weekly averages of NO_2_ and temperature from conception to delivery, contemporary with exposures to PM_2.5_ and its constituents.

### 2.5. Statistical Analysis

We applied the parametric G-computation combined with distributed lag nonlinear model (DLNM) to estimate the health associations of hypothetical interventions of reducing prenatal exposure to PM_2.5_ and its constituents on the cumulative risk of PTB [27]. We considered a series of interventions with different intensities. Specifically, we hypothesized that the weekly exposure levels of PM_2.5_ and its constituents would be reduced by a given percentage throughout pregnancy while assuming all other time-varying covariates remaining at their actual levels. The relative percentage of reductions were set at 10%, 20%, 40%, 50%, 60%, 80%, and 90%. The 40% reduction intervention was used as an example when illustrating the results as such reduction could reduce the PM_2.5_ levels to 15 μg/m^3^, approaching the WHO global air quality guidelines (AQGs 2021) [39].

Due to the strong correlations between the PM_2.5_ constituents, we examined each constituent separately. The cumulative risk difference (RD) was calculated to quantify the change in the cumulative probability of PTB up to week 36 (i.e., the threshold for PTB) attributed to a simultaneous intervention in each gestational week. Afterwards, we estimated the number of preventable PTBs under each hypothetical intervention scenario.

In brief, we conducted the hypothetical intervention analyses in four steps. First, we formatted the original data into a long-format structure and split the dataset based on gestational week at delivery. Second, we estimated the risk of PTB under the natural course (i.e., without interventions). Third, we estimated the risk of PTB under each hypothetical intervention strategy. Finally, we calculated the cumulative risk difference and the number of PTBs that could be prevented. More details can be found in Method S1.

To examine potential modifiers of the association, we carried out subgroup analyses stratified by infant sex and maternal age (<30 vs. ≥30 years).

To assess the robustness of our results, we performed several sensitivity analyses: (1) Infants with low birth weight (<2500 g) or macrosomia (≥4000 g) were excluded to reduce the potential influence of these birth weight extremes; (2) NO_2_ was removed from the models due to its moderate correlation with PM_2.5_ constituents, to assess the impact of collinearity; (3) PTB was redefined using 20 weeks of gestation as the lower threshold, and all primary analyses were repeated to evaluate the influence of gestational age definition; (4) Two-constituent models were constructed by simultaneously including two PM_2.5_ constituents in the same model to allow for mutual adjustment.

All statistical analyses were conducted using R version 4.4.1. A two-tailed *p* value of <0.05 defined statistical significance.

## 3. Results

### 3.1. Population Characteristics

Baseline characteristics of the study population are presented in Table 1. Among the 148,068 mother–child pairs included, 9757 (6.59%) births were classified as PTBs. The distribution of gestational weeks is also shown in Table S2. Compared to term births, preterm infants were more likely to be male, and their mothers were older at delivery and had a greater pre-pregnancy BMI. Regarding pregnancy conditions, mothers of PTB infants had higher rates of gestational hypertension and gestational diabetes compared to their counterparts.

### 3.2. Exposure Distributions and Correlations

The distributions of PM_2.5_, BC, OM, NH_4_^+^, NO_3_**^−^**, SO_4_^2−^, NO_2_, and temperature are shown in Tables S3 and S4. Overall, the concentrations were relatively stable throughout pregnancy, with median concentrations of 20.0 μg/m^3^ for PM_2.5_, 1.1 μg/m^3^ for BC, 5.2 μg/m^3^ for OM, 1.8 μg/m^3^ for NH_4_^+^, 2.3 μg/m^3^ for NO_3_**^−^**, 4.0 μg/m^3^ for SO_4_^2−^, 30.0 μg/m^3^ for NO_2_, and 25.0 °C for temperature. PM_2.5_ and its constituents exhibited strong positive correlations with each other, moderate positive correlations with NO_2_, and moderate negative correlations with temperature (Table S5).

### 3.3. Association of PM_2.5_ and Constituents with PTB

**Figure 1:**
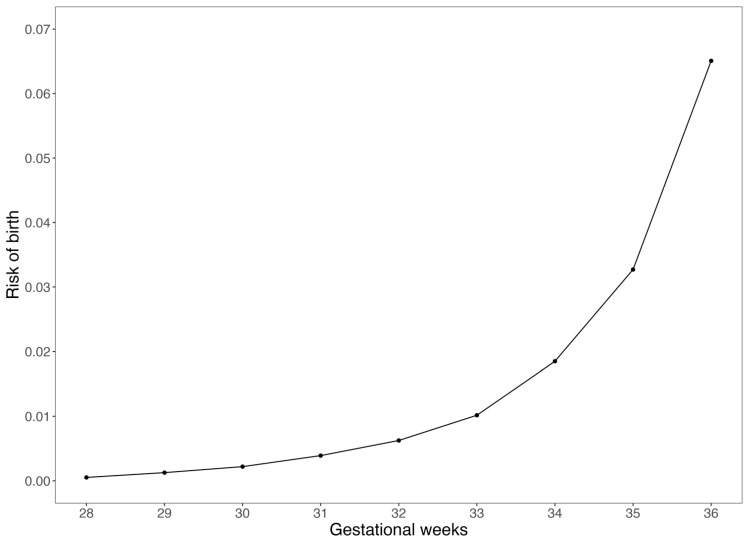
Cumulative risk of birth in gestational weeks 28–36 under natural course (without interventions).

**Figure 2:**
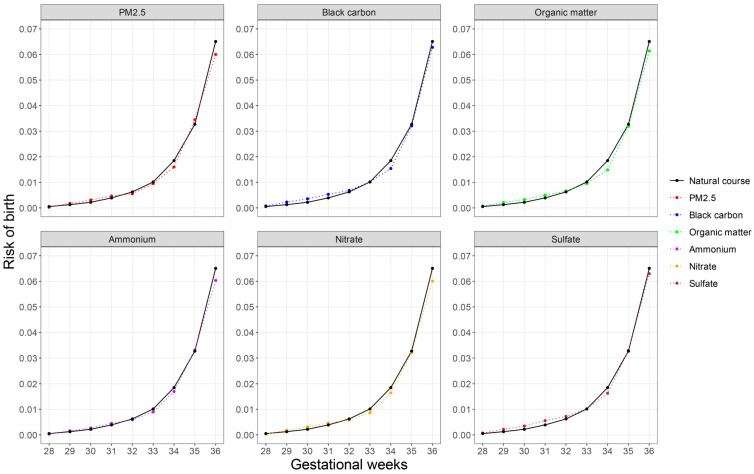
Cumulative risk of birth at gestational weeks 28–36 under natural course and 40% reduction intervention.

We observed that under the natural course (i.e., without interventions), the cumulative risk of PTB gradually increased with advancing gestational weeks. In particular, the increases in cumulative risk were relatively slow before gestational week 33, but accelerated thereafter and reached the peak at week 36 (Figure 1). Under all intervention scenarios, the cumulative risks of birth at 33–36 weeks of gestation (i.e., the risk of PTB) were lower than the risks under natural course. Such pattern was consistently observed for PM_2.5_ and all five constituents. (Figure 2, Tables S6–S12 and Figures S2–S7).

As shown in Table 2, hypothetical reductions in gestational exposure to PM_2.5_ and its constituents were associated with lower risks of PTB. For example, for the intervention of reducing PM_2.5_ by 40%, the cumulative risk of PTB was reduced by 5.08 per thousand births, corresponding to 752 preventable PTBs (7.71% of total PTBs) (Table 3). Such reductions in BC, OM, NH_4_^+^, NO_3_**^−^**, and SO_4_^2−^ were associated with a risk difference of −2.29, −3.70, −4.74, −5.00, and −2.11 per thousand births, respectively, corresponding to 339, 548, 702, 740, and 312 preventable PTBs (Table 2 and Table 3). These figures represent 3.47%, 5.62%, 7.19%, 7.58%, and 3.20% of the total PTBs, respectively. In addition, as the intervention intensity increased, the cumulative risk difference also increased, indicating a dose–response relationship (Table 2).

In stratified analyses, we found that the reductions in PM_2.5_ and its constituents were consistently with reduced risks of PTB in subgroups by infant sex and maternal age (Table S13). We did not observe significant effect modification by any of the examined variables.

### 3.4. Sensitivity Analysis

Overall, the sensitivity analyses yielded results that were consistent with our main findings. Specifically, exclusion of infants with low birth weight or macrosomia did not significantly alter the cumulative risk of PTB (Tables S14 and S15). Excluding NO_2_ from the models also did not lead to notable changes in the results (Tables S14 and S16). Extremely early PTBs (<28 weeks) were relatively rare in our study population (Table S17). When PTB was redefined using 20 weeks of gestation as the lower threshold, the estimated associations remained materially unchanged compared with the primary analysis (Tables S14 and S18). For the two-constituent models, although the results differed slightly from those of the main analysis, they did not substantially alter the overall pattern of associations (Table S19).

## 4. Discussion

Based on a large birth cohort, our study found that hypothetical reductions in the concentrations of all five constituents of PM_2.5_ during pregnancy were associated with decreased risks of PTB. Moreover, the decreases in the PTB risks increased with the increasing intensity of the interventions, demonstrating a dose–response relationship.

As far as we are aware, no previous study has investigated the link between gestational exposure to PM_2.5_ constituents and PTB using a hypothetical intervention design. Our results demonstrated that hypothetical interventions of reducing PM_2.5_ constituents’ exposure during pregnancy were associated with a reduced risk of PTB. Stronger associations were observed for NO_3_**^−^** and NH_4_^+^ than for other constituents. Consistent with these observations, a nested case–control study in California, USA [40], found that NO_3_**^−^** and NH_4_^+^ showed the most pronounced positive relationships among PM_2.5_ constituents. In addition, a multi-city Chinese cohort study further identified associations between gestational exposure to NH_4_^+^, NO_3_**^−^**, and SO_4_^2^**^−^** and higher PTB risk, highlighting NH_4_^+^ as the constituent with the strongest associations [24]. A prospective cohort study of 409,037 newborns in the USA also found that exposure to BC, OM, SO_4_^2^**^−^**, NH_4_^+^, and NO_3_**^−^** throughout pregnancy was associated with the occurrence of PTB, among which BC had the most significant associations [23]. Similar associations were identified in a large-scale birth cohort study covering 336 Chinese cities [21], whereas a retrospective cohort study in Atlanta, USA, failed to detect significant associations between NH_4_^+^ or NO_3_**^−^** exposure and PTB [41].

Taken together, current findings of the associations between PM_2.5_ constituents and PTB are still inconsistent. Differences in research design, population demographics, exposure measurement techniques, and statistical analyses may account for these inconsistent findings. It should be emphasized that most previous studies treated PTB as a binary outcome and mainly used traditional statistical methods such as logistic regression, which may have introduced immortal time bias (ITB) [42,43]. In our study, we treated PTB as a time-to-event survival outcome and used DLNM to control the lagged effects of exposure, which could help to reduce the influence of ITB. More importantly, we adopted a hypothetical intervention study design based on the parametric G-computation, which could simulate the causal inference framework of experimental study. By incorporating Monte Carlo simulations, we constructed multiple hypothetical scenarios of reducing prenatal exposure to air pollutants. This approach enabled us to directly estimate the risk of outcomes (i.e., PTB) under different exposure trajectories.

Another strength of the hypothetical intervention approach is it could directly provide the estimates of health benefits achieved through implementing the hypothetical interventions. In Foshan, for example, a 40% reduction in PM_2.5_ exposure during pregnancy was linked to 752 preventable PTB cases when compared with the natural course. Such estimates are relatively more intuitive to stakeholders involved in public health decision-making.

The precise biological mechanisms underlying the PM_2.5_-induced adverse impact on PTB remain unclear. Previous research had suggested that PM_2.5_ may contribute to PTB through mechanisms such as oxidative stress, lipid peroxidation, DNA adducts formation, and disruption of endocrine regulation (e.g., pituitary–adrenocortical–placental system) [44,45,46,47]. To be noted, current research on the specific mechanistic roles of individual PM_2.5_ constituents is even more scarce. Evidence from existing research suggests that BC deposits on the fetal placental surface may provoke inflammation and immune modifications, thereby increasing the likelihood of negative birth outcomes [48]. Some other studies also indicated that prenatal exposure to NO_3_**^−^**, NH_4_^+^, and SO_4_^2^**^−^** was associated with elevated systemic inflammation [49], while OM may reduce antioxidant enzyme activity [24], both of which could contribute to PTB [50]. A recent study also highlighted that combined exposure to PM_2.5_ constituents might further promote PTB by disrupting the oxidative phosphorylation pathway [51]. Further research is warranted to explore the biological pathways of specific PM_2.5_ constituents in order to elucidate their toxic mechanisms.

Our study benefited from a cohort study design, individual-level exposure assessment, and the employment of hypothetical intervention strategies based on the parametric G-computation as discussed above. However, several limitations should be acknowledged. First, we only focused on five major PM_2.5_ constituents, precluding an assessment on specific elements (e.g., As, Cd, Co). Second, nonparametric bootstrap with 100 replications were used to estimate confidence intervals, primarily attributable to computational constraints. Due to the limited number of bootstrap samples, the constructed empirical distribution may inadequately approximate the true sampling distribution of the statistic, potentially leading to instability in the estimation of confidence intervals [52]. Future studies with enhanced computational resources could explore increasing the number of bootstrap iterations. Third, the exposure assessment did not account for individuals’ activity pattern and only considered outdoor air pollution levels, which might have resulted in exposure misclassification. Nevertheless, the misclassification was probably nondifferential, and therefore unlikely to have significantly biased the results.

## 5. Conclusions

In conclusion, the present study revealed that reducing gestational exposure to all five major PM_2.5_ constituents (BC, OM, NO_3_**^−^**, NH_4_^+^, SO_4_^2^**^−^**) was associated with a lower risk of PTB. Our study provides novel epidemiological evidence on the differential impacts of PM_2.5_ constituents on PTB, highlighting the importance of the continuing efforts to reduce PM_2.5_ exposure, especially certain constituents.

## Figures and Tables

**Table 1:** General characteristics of study participants.

Characteristics	All Participants	Preterm Birth	Term Birth	*p * ^a^
Number of participants	148,068	9757	138,311	
Maternal age (years)	29.52 (4.77)	30.52 (5.14)	29.45 (4.73)	<0.001
Pre-pregnancy BMI (kg/m^2^) ^b^	22.64 (3.45)	22.94 (3.70)	22.62 (3.43)	<0.001
Employment status				0.032
Employed	120,050 (81.1%)	7830 (80.3%)	112,220 (81.1%)	
Unemployed	28,018 (18.9%)	1927 (19.7%)	26,091 (18.9%)	
Education ^c^				0.010
High	75,865 (51.2%)	4872 (49.9%)	70,993 (51.3%)	
Medium	35,040 (23.7%)	2322 (23.8%)	32,718 (23.7%)	
Low	37,163 (25.1%)	2563 (26.3%)	34,600 (25.0%)	
Local household registration				<0.001
Yes	67,621 (45.7%)	4790 (49.1%)	62,831 (45.4%)	
No	80,447 (54.3%)	4967 (50.9%)	75,480 (54.6%)	
Gestational hypertension				<0.001
Yes	2007 (1.4%)	245 (2.5%)	1762 (1.3%)	
No	146,061 (98.6%)	9512 (97.5%)	136,549 (98.7%)	
Gestational diabetes mellitus				0.001
Yes	15,368 (10.4%)	1110 (11.4%)	14,258 (10.3%)	
No	132,700 (89.6%)	8647 (88.6%)	124,053 (89.7%)	
Infant sex				<0.001
Male	79,393 (53.6%)	5613 (57.5%)	73,780 (53.3%)	
Female	68,675 (46.4%)	4144 (42.5%)	64,531 (46.7%)	
Season of conception				<0.001
Spring	34,573 (23.3%)	2338 (24.0%)	32,235 (23.3%)	
Summer	34,779 (23.5%)	2284 (23.4%)	32,495 (23.5%)	
Autumn	40,621 (27.4%)	2807 (28.8%)	37,814 (27.3%)	
Winter	38,095 (25.7%)	2328 (23.9%)	35,767 (25.9%)	

Data are presented as mean (standard deviation) and number (percentage) for continuous and categorical variables, respectively. ^a^ *p* value for comparison between preterm birth and term birth. ^b^ BMI: body mass index. ^c^ High: college or above; Medium: high school or equivalent technical school; Low: middle school or lower.

**Table 2:** Cumulative risk difference and 95% CI per thousand births at gestational week 36 under different intervention strategies.

Reduction (%)	PM_2.5_ and Its Constituents					
	PM_2.5_	BC	OM	NH_4_^+^	NO_3_^−^	SO_4_^2−^
10	−1.37 (−4.24, 1.58)	−0.62 (−3.28, 2.27)	−0.99 (−3.67, 1.89)	−1.21 (−4.53, 1.61)	−1.28 (−4.53, 0.96)	−0.56 (−3.50, 2.88)
20	−2.67 (−8.53, 3.59)	−1.21 (−6.56, 4.21)	−1.93 (−9.05, 3.42)	−2.40 (−8.31, 1.86)	−2.54 (−7.94, 1.25)	−1.10 (−7.72, 5.38)
40	−5.08 (−20.54, 8.94)	−2.29 (−12.39, 9.01)	−3.70 (−15.37, 5.14)	−4.74 (−14.93, 4.02)	−5.00 (−15.72, 5.87)	−2.11 (−18.37, 10.43)
50	−6.19 (−24.69, 9.00)	−2.78 (−18.43, 10.37)	−4.52 (−20.80, 8.63)	−5.88 (−19.71, 6.09)	−6.19 (−20.09, 5.34)	−2.59 (−19.01, 12.27)
60	−7.24 (−26.12, 10.33)	−3.23 (−18.36, 18.66)	−5.30 (−23.80, 8.96)	−7.01 (−24.44, 8.08)	−7.37 (−27.29, 6.31)	−3.04 (−24.70, 17.09)
80	−9.14 (−31.66, 17.82)	−4.04 (−31.90, 19.85)	−6.73 (−32.06, 17.38)	−9.21 (−26.20, 6.94)	−9.66 (−32.75, 7.15)	−3.87 (−29.93, 24.98)
90	−9.99 (−36.78, 25.89)	−4.39 (−24.43, 23.32)	−7.38 (−29.75, 16.06)	−10.30 (−35.45, 10.41)	−10.82 (−32.43, 11.14)	−4.25 (−29.61, 18.31)

Abbreviations: CI: confidence interval; PM_2.5_: particulate matter with aerodynamic diameter less than 2.5 µm; BC: black carbon; OM: organic matter; NH_4_^+^: ammonium; NO_3_^−^: nitrate; SO_4_^2−^: sulfate. Cumulative risk differences were calculated by comparing each intervention strategies with no intervention (natural course).

**Table 3:** Number of preventable cases of preterm birth under different intervention strategies.

Reduction (%)	PM_2.5_ and Its Constituents					
	PM_2.5_	BC	OM	NH_4_^+^	NO_3_^−^	SO_4_^2−^
10	203	92	147	179	190	83
20	395	179	286	355	376	163
40	752	339	548	702	740	312
50	917	412	669	871	917	383
60	1072	478	785	1038	1091	450
80	1353	598	996	1364	1430	573
90	1479	650	1093	1525	1602	629

Abbreviations: PM_2.5_: particulate matter with aerodynamic diameter less than 2.5 µm; BC: black carbon; OM: organic matter; NH_4_^+^: ammonium; NO_3_^−^: nitrate; SO_4_^2−^: sulfate.

## Data Availability

The data presented in this study are available on request from the corresponding author due to privacy issues.

## Supplementary Materials

The following supporting information can be downloaded at: https://www.mdpi.com/article/10.3390/toxics14030233/s1, Method S1: Detailed information on hypothetical intervention analyses. Table S1: General characteristics of the original cohort and analytical cohort. Table S2: Distribution of gestational weeks. Table S3: Distributions of PM_2.5_ and its constituents by gestational week. Table S4: Distributions of NO_2_ and ambient temperature by gestational week. Table S5: Correlation coefficients between air pollutants and ambient temperature during pregnancy. Table S6: Cumulative risk of birth at gestational weeks 28–36 under natural course and 10% reduction intervention. Table S7: Cumulative risk of birth at gestational weeks 28–36 under natural course and 20% reduction intervention. Table S8: Cumulative risk of birth at gestational weeks 28–36 under natural course and 40% reduction intervention. Table S9: Cumulative risk of birth at gestational weeks 28–36 under natural course and 50% reduction intervention. Table S10: Cumulative risk of birth at gestational weeks 28–36 under natural course and 60% reduction intervention. Table S11: Cumulative risk of birth at gestational weeks 28–36 under natural course and 80% reduction intervention. Table S12: Cumulative risk of birth at gestational weeks 28–36 under natural course and 90% reduction intervention. Table S13: Cumulative risk difference and 95% CI per thousand births at gestational week 36 under 40% reduction in PM_2.5_ and its constituents’ intervention in stratified analysis. Table S14: Cumulative risk difference and 95% CI per thousand births at gestational week 36 under 20% reduction intervention in sensitivity analyses (1), (2) and (3). Table S15: Cumulative risk of birth at gestational weeks 28–36 under natural course and 20% reduction intervention in sensitivity analysis excluding infants with low birth weight or macrosomia. Table S16: Cumulative risk of birth at gestational weeks 28–36 under natural course and 20% reduction in sensitivity analysis excluding nitrogen dioxide in the models. Table S17: Distribution of gestational weeks in sensitivity analysis redefining preterm birth using a 20-week threshold. Table S18: Cumulative risk of birth at gestational weeks 24–36 under natural course and 20% reduction in sensitivity analysis using a 20-week definition of preterm birth. Table S19: Cumulative risk difference and 95% CI per thousand births at gestational week 36 under 20% reduction intervention in two-constituent models. Figure S1: The process of participant selection. Figure S2: Cumulative risk of birth at gestational weeks 28–36 under natural course and 10% reduction intervention. Figure S3: Cumulative risk of birth at gestational weeks 28–36 under natural course and 20% reduction intervention. Figure S4: Cumulative risk of birth at gestational weeks 28–36 under natural course and 50% reduction intervention. Figure S5: Cumulative risk of birth at gestational weeks 28–36 under natural course and 60% reduction intervention. Figure S6: Cumulative risk of birth at gestational weeks 28–36 under natural course and 80% reduction intervention. Figure S7: Cumulative risk of birth at gestational weeks 28–36 under natural course and 90% reduction intervention.

## Abbreviations

PTB	Preterm birth
PM_2.5_	Fine particulate matter
BC	Black carbon
OM	Organic matter
NO_3_**^−^**	Nitrate
NH_4_^+^	Ammonium
SO_4_^2^**^−^**	Sulfate
TAP	Tracking air pollution in China
LMP	Last menstrual period
BMI	Body mass index
NO_2_	Nitrogen dioxide
DLNM	Distributed lag nonlinear model
AQGs	Global air quality guidelines
RD	Risk difference
ITB	Immortal time bias
