# Supplementary material for: Associations of Gestational Exposure to Fine Particulate Matter Constituents with Preterm Birth: A Birth Cohort-Based Hypothetical Intervention Study
Source: Toxics. 2026 Mar 9;14(3):233. doi: 10.3390/toxics14030233 (PMC13030305; doi:10.3390/toxics14030233)
Supplement: Supplementary file 1 [file toxics-14-00233-s001.zip › toxics-4164326-supplementary.pdf]

Supplementary material

Associations of gestational exposure to fine particulate matter constituents with preterm birth: A birth cohort-based hypothetical intervention study

Yonggui Gao [1,†], Rui Qian [2,†], Xinyue Li [1], Sheng Qiu [3], Zijun Yang [1], Saijun Huang [4], Pengzhen Hu [4], Yin Yang [1], Hualiang Lin [1], Xi Su [4], Qingmei Lin [4,*], Zilong Zhang [1,*]

1. Department of Epidemiology, School of Public Health, Sun Yat-sen University, Guangzhou 510080, China

2. Statistical Information Center for Health and Family Planning of Foshan, Foshan 528000, China

3. Nanhai Maternal and Children's Hospital of Guangzhou University of Chinese Medicine, Foshan 528000, China

4. Foshan Women and Children Hospital Affiliated to Guangdong Medical University, Foshan 528315, China

† These two authors contributed equally to this work.

* Correspondence to: Dr. Qingmei Lin, Foshan Women and Children Hospital Affiliated to Guangdong Medical University, Foshan 528315, China. E-mail: linqm01@mail.fsfy.com; or Dr. Zilong Zhang, Department of Epidemiology, School of Public Health, Sun Yat-sen University, Guangzhou 510080, China. E-mail: zhangzilong@mail.sysu.edu.cn.

Contents

Method S1: Detailed information on hypothetical intervention analyses.

Table S1: General characteristics of the original cohort and analytical cohort.

Table S2: Distribution of gestational weeks.

Table S3: Distributions of $PM_{2.5}$ and its constituents by gestational week.

Table S4: Distributions of NO_2 and ambient temperature by gestational week.

Table S5: Correlation coefficients between air pollutants and ambient temperature during pregnancy.

Table S6: Cumulative risk of birth at gestational weeks 28-36 under natural course and 10% reduction intervention.

Table S7: Cumulative risk of birth at gestational weeks 28-36 under natural course and 20% reduction intervention.

Table S8: Cumulative risk of birth at gestational weeks 28-36 under natural course and 40% reduction intervention.

Table S9: Cumulative risk of birth at gestational weeks 28-36 under natural course and 50% reduction intervention.

Table S10: Cumulative risk of birth at gestational weeks 28-36 under natural course and 60% reduction intervention.

Table S11: Cumulative risk of birth at gestational weeks 28-36 under natural course and 80% reduction intervention.

Table S12: Cumulative risk of birth at gestational weeks 28-36 under natural course and 90% reduction intervention.

Table S13: Cumulative risk difference and 95% CI per thousand births at gestational week 36 under 40% reduction in $PM_{2.5}$ and its constituents' intervention in stratified analysis.

Table S14: Cumulative risk difference and 95% CI per thousand births at gestational week 36 under 20% reduction intervention in sensitivity analyses (1), (2) and (3).

Table S15: Cumulative risk of birth at gestational weeks 28-36 under natural course

and 20% reduction intervention in sensitivity analysis excluding infants with low birth weight or macrosomia.

Table S16: Cumulative risk of birth at gestational weeks 28-36 under natural course and 20% reduction in sensitivity analysis excluding nitrogen dioxide in the models.

Table S17: Distribution of gestational weeks in sensitivity analysis redefining preterm birth using a 20-week threshold.

Table S18: Cumulative risk of birth at gestational weeks 24-36 under natural course and 20% reduction in sensitivity analysis using a 20-week definition of preterm birth.

Table S19: Cumulative risk difference and 95% CI per thousand births at gestational week 36 under 20% reduction intervention in two-constituent models.

Figure S1: The process of participant selection.

Figure S2: Cumulative risk of birth at gestational weeks 28-36 under natural course and 10% reduction intervention.

Figure S3: Cumulative risk of birth at gestational weeks 28-36 under natural course and 20% reduction intervention.

Figure S4: Cumulative risk of birth at gestational weeks 28-36 under natural course and 50% reduction intervention.

Figure S5: Cumulative risk of birth at gestational weeks 28-36 under natural course and 60% reduction intervention.

Figure S6: Cumulative risk of birth at gestational weeks 28-36 under natural course and 80% reduction intervention.

Figure S7: Cumulative risk of birth at gestational weeks 28-36 under natural course and 90% reduction intervention.

Method S1: Detailed information on hypothetical intervention analyses.

In Step 1, we first formatted the dataset, including: 1A) Transforming the original wide-format data into a long-format structure, where each row represents an individual-week observation during pregnancy; 1B) Splitting the long-format data into nine separate datasets according to the study design, each corresponding to a specific PTB risk set (i.e., follow-up until gestational week 28, 29, 30, up to week 36).

In Step 2, we estimated the risk of PTB under the natural course (i.e., without interventions): 2A) For each risk set, we fitted a DLNM using the observed weekly exposure history during pregnancy. The specific model was expressed as:

$$g(E[Y_{ij}]) = \beta_0 + \sum_{t=1}^{j} f(X_{it}\,; \alpha_t) + \sum_{t=1}^{j} f(NO2_{it}\,; \delta_t) + \sum_{t=1}^{j} f(TMP_{it}\,; \xi_t) + z_i^T \gamma,$$

where $g()$ denotes the link function family, Y_{ij} represents the delivery indicator for individual i in risk set j, β_0 is the intercept, f denotes the basis function of the independent variable, X represents the exposure variable ($PM_{2.5}$ and its constituents), and α_t, δ_t, and ξ_t are the regression coefficients for the exposure variable, NO_2, and temperature at gestational week t, respectively. Additionally, z_i^T denotes the vector of covariates associated with regression coefficients γ, including maternal age, pre-pregnancy BMI, and other variables specified in Section 2.4 (Covariates).

We assumed non-linear exposure–response relationships for exposure, NO_2, and temperature, and applied a natural spline function with four degrees of freedom to capture both exposure–response and lag effects. All covariates described previously were included in the models; 2B) for each risk set, we predicted the conditional probability of not delivering for each pregnant woman in risk set j (i.e., weeks 28, 29, 30, up to 36) under the natural course; 2C) we then combined the conditional probabilities from the nine risk sets into a long-format dataset and calculated the cumulative probability of not delivering by gestational week j for each participant. This was achieved by multiplying the conditional probabilities of not delivering up to week j, and then converting it into the cumulative probability of delivery (i.e., the risk of PTB); 2D) Finally, we estimated the overall PTB risk under the natural course for

each gestational week j by averaging the cumulative delivery probabilities across all participants.

In Step 3, we estimated the risk of PTB under each hypothetical intervention strategy: 3A) Based on the long-format data generated in Step 1A), we simulated reducing the exposure variable ($PM_{2.5}$ and its constituents) for all pregnant women in the first gestational week by a fixed percentage of the original exposure level (i.e., 10%, 20%, 40%, 50%, 60%, 80%, and 90%) while keeping the exposure levels of subsequent gestational weeks and other time-varying covariates (NO_2 and temperature) unchanged as their actual observed values. We created this simulated long-format dataset and repeated the data-splitting procedure described in Step 1B), resulting in nine separate datasets corresponding to each risk set; 3B) using the regression coefficients obtained from the model in Step 2A), we predicted the conditional probability of not delivering under the simulated intervention scenario for each pregnant woman in each risk set [similar to Step 2B)]; 3C) following the same approach as in Step 2C), we calculated the cumulative probability of delivery (i.e., the risk of PTB) under the simulated intervention scenario for each participant by multiplying the conditional probabilities of not delivering up to week j; 3D) consistent with Step 2D), we estimated the overall PTB risk under each simulated intervention scenario for each gestational week j by averaging the cumulative delivery probabilities across all participants; 3E) we repeated the above steps for each gestational week, simulating the intervention at each specific week of pregnancy. This process yielded a total of 36 cumulative risk curves, each representing the cumulative risk of PTB given a hypothetical intervention at a specific gestational week.

In Step 4, we calculated the cumulative risk difference and the number of PTBs that could be prevented: 4A) We compared each of the 36 cumulative risk curves generated in Step 3E) under different intervention scenarios with the cumulative risk curve under the natural course estimated in Step 2. Specifically, for each intervention curve, we used the cumulative risk at each gestational week j (i.e., weeks 28, 29, 30, up to 36) and subtracted the corresponding cumulative risk under the natural course, allowing us to quantify how an intervention in a single gestational week altered the

cumulative risk of PTB (i.e., the cumulative risk difference); 4B) we summed these weekly cumulative risk differences to calculate the overall cumulative risk difference for each intervention scenario. Multiplying the cumulative risk difference by the cohort size provided an estimate of the number of PTBs that could be prevented under each simulated intervention; 4C) to calculate the 95% confidence intervals (CIs), we performed a nonparametric bootstrap with 100 replications (based on the 2.5th and 97.5th percentiles of the bootstrapped distribution) using the “boot” package.

Table S1: General characteristics of the original cohort and analytical cohort.

Characteristics	Original cohort	Analytical cohort
Number of participants	322,481	148,068
Maternal age (years)	29.44 (4.76)	29.52 (4.77)
Pre-pregnancy BMI (kg/m^2) [a]	22.79 (3.48)	22.64 (3.45)
Employment status		
Employed	184,698 (84.5%)	120,050 (81.1%)
Unemployed	33,999 (15.5%)	28,018 (18.9%)
Missing	103,784	-
Education [b]		
High	96,829 (49.5%)	75,865 (51.2%)
Medium	47,416 (24.2%)	35,040 (23.7%)
Low	51,443 (26.3%)	37,163 (25.1%)
Missing	126,793	-
Local household registration		
Yes	153,836 (53.4%)	67,621 (45.7%)
No	134,177 (46.6%)	80,447 (54.3%)
Missing	34,468	-
Gestational hypertension		
Yes	3,832 (1.4%)	2,007 (1.4%)
No	271,578 (98.6%)	146,061 (98.6%)
Missing	47,071	-
Gestational diabetes mellitus		
Yes	21,409 (6.6%)	15,368 (10.4%)
No	301,072 (93.4%)	132,700 (89.6%)
Infant sex		
Male	171,579 (53.2%)	79,393 (53.6%)
Female	150,902 (46.8%)	68,675 (46.4%)
Season of conception		
Spring	73,380 (22.8%)	34,573 (23.3%)
Summer	75,626 (23.5%)	34,779 (23.5%)
Autumn	88,460 (27.4%)	40,621 (27.4%)
Winter	85,015 (26.4%)	38,095 (25.7%)

Data are presented as mean (standard deviation) and number (percentage) for continuous and categorical variables, respectively.

[a] BMI: body mass index.

[b] High: college or above; Medium: high school or equivalent technical school; Low: middle school or lower.

Table S2: Distribution of gestational weeks.

Gestational week	Number of deliveries (%)
28	78 (0.05%)
29	110 (0.07%)
30	138 (0.09%)
31	254 (0.17%)
32	348 (0.24%)
33	584 (0.40%)
34	1,248 (0.84%)
35	2,133 (1.44%)
36	4,864 (3.29%)
⩾ 37	138,311 (93.41%)

Table S3: Distributions of $PM_{2.5}$ and its constituents by gestational week.

Gestational week	$PM_{2.5}$ (μg/m^3)				BC (μg/m^3)				OM (μg/m^3)				NH_4^+ (μg/m^3)				NO_3^- (μg/m^3)				SO_4^{2-} (μg/m^3)			
	Mean (SD)	Median	P25	P75	Mean (SD)	Median	P25	P75	Mean (SD)	Median	P25	P75	Mean (SD)	Median	P25	P75	Mean (SD)	Median	P25	P75	Mean (SD)	Median	P25	P75
1	22.33 (12.36)	20.48	12.64	29.63	1.18 (0.61)	1.12	0.68	1.59	5.78 (3.13)	5.42	3.18	7.86	2.32 (1.72)	1.89	0.99	3.05	3.23 (2.66)	2.51	1.23	4.17	4.37 (2.27)	4.08	2.52	5.90
2	22.24 (12.24)	20.38	12.60	29.53	1.18 (0.61)	1.12	0.68	1.58	5.76 (3.10)	5.40	3.16	7.82	2.31 (1.72)	1.88	1.00	3.04	3.23 (2.65)	2.51	1.24	4.15	4.36 (2.25)	4.08	2.53	5.87
3	22.18 (12.09)	20.39	12.65	29.53	1.18 (0.60)	1.12	0.68	1.58	5.74 (3.07)	5.41	3.17	7.79	2.31 (1.70)	1.89	1.01	3.02	3.22 (2.62)	2.50	1.25	4.15	4.35 (2.23)	4.08	2.54	5.87
4	22.20 (12.11)	20.38	12.65	29.54	1.18 (0.60)	1.12	0.69	1.58	5.75 (3.08)	5.41	3.17	7.80	2.31 (1.70)	1.89	1.01	3.03	3.23 (2.63)	2.51	1.25	4.17	4.35 (2.23)	4.06	2.53	5.87
5	22.25 (12.08)	20.49	12.74	29.68	1.18 (0.60)	1.13	0.69	1.58	5.77 (3.06)	5.45	3.22	7.83	2.32 (1.71)	1.89	1.01	3.03	3.24 (2.63)	2.51	1.26	4.17	4.35 (2.22)	4.08	2.55	5.88
6	22.23 (12.03)	20.53	12.67	29.75	1.18 (0.60)	1.13	0.69	1.58	5.77 (3.05)	5.47	3.20	7.82	2.33 (1.71)	1.89	1.01	3.05	3.24 (2.63)	2.51	1.26	4.19	4.36 (2.22)	4.09	2.54	5.88
7	22.14 (11.8	20.42	12.75	29.61	1.18 (0.59)	1.12	0.70	1.57	5.74 (3.02)	5.42	3.23	7.77	2.32 (1.70)	1.88	1.02	3.04	3.23 (2.61)	2.50	1.26	4.19	4.34 (2.21)	4.06	2.56	5.86

	6)																							
8	22.09 (11.84)	20.32	12.72	29.57	1.17 (0.59)	1.12	0.69	1.56	5.73 (3.01)	5.40	3.21	7.77	2.31 (1.69)	1.89	1.01	3.04	3.22 (2.60)	2.49	1.26	4.19	4.33 (2.20)	4.05	2.55	5.84
9	22.05 (11.81)	20.28	12.64	29.62	1.17 (0.59)	1.11	0.69	1.56	5.72 (3.01)	5.38	3.19	7.78	2.31 (1.70)	1.87	1.00	3.06	3.23 (2.61)	2.48	1.24	4.20	4.32 (2.20)	4.05	2.53	5.82
10	21.99 (11.73)	20.22	12.63	29.55	1.17 (0.58)	1.11	0.69	1.56	5.71 (2.99)	5.37	3.19	7.76	2.31 (1.69)	1.89	1.00	3.06	3.23 (2.60)	2.49	1.24	4.24	4.30 (2.19)	4.03	2.53	5.82
11	21.91 (11.70)	20.14	12.56	29.51	1.16 (0.58)	1.11	0.68	1.55	5.69 (3.00)	5.34	3.16	7.73	2.30 (1.69)	1.87	0.99	3.06	3.22 (2.60)	2.47	1.23	4.23	4.29 (2.19)	4.02	2.51	5.79
12	21.94 (11.69)	20.10	12.55	29.62	1.16 (0.58)	1.11	0.68	1.56	5.69 (3.00)	5.34	3.16	7.76	2.31 (1.69)	1.86	0.99	3.07	3.22 (2.59)	2.47	1.22	4.24	4.30 (2.20)	4.00	2.51	5.82
13	21.86 (11.72)	19.92	12.38	29.64	1.16 (0.59)	1.09	0.68	1.55	5.68 (3.02)	5.28	3.11	7.75	2.30 (1.70)	1.85	0.97	3.06	3.20 (2.59)	2.45	1.20	4.26	4.28 (2.22)	3.96	2.47	5.81
14	21.82 (11.78)	19.81	12.27	29.65	1.16 (0.59)	1.09	0.67	1.55	5.67 (3.04)	5.25	3.07	7.76	2.30 (1.70)	1.83	0.96	3.09	3.20 (2.60)	2.42	1.18	4.28	4.27 (2.23)	3.94	2.45	5.80
15	21.76 (11.79)	19.65	12.17	29.75	1.15 (0.59)	1.08	0.66	1.55	5.65 (3.05)	5.19	3.05	7.77	2.29 (1.70)	1.83	0.94	3.10	3.19 (2.59)	2.42	1.16	4.27	4.27 (2.25)	3.92	2.43	5.81

16	21.70 (11.81)	19.66	12.03	29.66	1.15 (0.59)	1.08	0.66	1.55	5.62 (3.05)	5.20	3.00	7.72	2.29 (1.72)	1.82	0.93	3.09	3.18 (2.61)	2.40	1.14	4.25	4.25 (2.26)	3.92	2.39	5.80
17	21.63 (11.82)	19.44	12.00	29.70	1.15 (0.60)	1.07	0.65	1.55	5.61 (3.06)	5.13	2.98	7.74	2.28 (1.71)	1.80	0.93	3.08	3.16 (2.59)	2.38	1.13	4.23	4.24 (2.26)	3.88	2.38	5.80
18	21.55 (11.78)	19.36	11.98	29.58	1.14 (0.60)	1.06	0.65	1.54	5.59 (3.06)	5.09	2.97	7.71	2.27 (1.70)	1.79	0.93	3.05	3.14 (2.58)	2.36	1.12	4.20	4.23 (2.26)	3.88	2.38	5.78
19	21.39 (11.66)	19.27	11.84	29.40	1.13 (0.59)	1.06	0.64	1.53	5.54 (3.03)	5.06	2.94	7.64	2.25 (1.69)	1.78	0.92	3.02	3.11 (2.56)	2.34	1.11	4.17	4.20 (2.25)	3.84	2.36	5.74
20	21.33 (11.60)	19.25	11.88	29.24	1.13 (0.59)	1.05	0.65	1.52	5.52 (3.01)	5.03	2.95	7.57	2.23 (1.67)	1.78	0.91	3.01	3.09 (2.53)	2.34	1.11	4.15	4.19 (2.23)	3.83	2.36	5.70
21	21.30 (11.62)	19.23	11.89	29.13	1.13 (0.59)	1.05	0.65	1.52	5.52 (3.02)	5.04	2.96	7.56	2.23 (1.68)	1.77	0.92	2.97	3.08 (2.54)	2.33	1.11	4.10	4.18 (2.24)	3.83	2.37	5.68
22	21.25 (11.61)	19.15	11.88	29.13	1.13 (0.59)	1.05	0.65	1.51	5.51 (3.02)	5.01	2.95	7.54	2.21 (1.67)	1.75	0.92	2.95	3.06 (2.53)	2.30	1.12	4.06	4.17 (2.23)	3.81	2.36	5.67
23	21.19 (11.55)	19.07	11.88	28.93	1.12 (0.59)	1.04	0.64	1.51	5.49 (3.01)	4.99	2.95	7.51	2.20 (1.66)	1.73	0.92	2.93	3.04 (2.51)	2.28	1.12	4.05	4.16 (2.23)	3.80	2.36	5.64
24	21.18 (11.5	19.16	11.98	28.85	1.12 (0.58)	1.05	0.65	1.50	5.49 (3.00)	5.00	2.97	7.47	2.20 (1.65)	1.74	0.92	2.92	3.04 (2.50)	2.29	1.12	4.03	4.16 (2.22)	3.81	2.38	5.63

	1)																							
25	21.14 (11.42)	19.16	12.02	28.71	1.12 (0.58)	1.05	0.65	1.50	5.47 (2.97)	5.02	2.98	7.42	2.19 (1.64)	1.72	0.93	2.90	3.02 (2.48)	2.27	1.13	3.99	4.15 (2.21)	3.83	2.38	5.61
26	21.18 (11.39)	19.19	12.09	28.68	1.12 (0.58)	1.05	0.66	1.49	5.49 (2.97)	5.03	3.02	7.42	2.19 (1.63)	1.72	0.94	2.89	3.01 (2.47)	2.27	1.15	3.98	4.16 (2.20)	3.83	2.40	5.61
27	21.11 (11.29)	19.12	12.23	28.46	1.12 (0.57)	1.05	0.66	1.48	5.48 (2.95)	5.03	3.06	7.36	2.18 (1.63)	1.71	0.95	2.86	3.00 (2.45)	2.25	1.16	3.95	4.15 (2.18)	3.82	2.43	5.58
28	21.12 (11.26)	19.21	12.25	28.45	1.12 (0.57)	1.05	0.66	1.49	5.48 (2.95)	5.03	3.06	7.37	2.17 (1.61)	1.72	0.95	2.85	2.99 (2.43)	2.26	1.17	3.93	4.15 (2.18)	3.83	2.43	5.57
29	21.05 (11.20)	19.14	12.20	28.46	1.12 (0.57)	1.05	0.66	1.48	5.47 (2.93)	5.02	3.05	7.38	2.16 (1.60)	1.71	0.95	2.83	2.97 (2.41)	2.25	1.17	3.91	4.14 (2.17)	3.81	2.42	5.58
30	21.04 (11.17)	19.21	12.22	28.29	1.12 (0.57)	1.05	0.66	1.48	5.47 (2.93)	5.03	3.07	7.34	2.15 (1.59)	1.71	0.95	2.82	2.96 (2.40)	2.26	1.16	3.90	4.14 (2.17)	3.82	2.43	5.54
31	21.03 (11.19)	19.14	12.21	28.32	1.12 (0.57)	1.05	0.66	1.48	5.47 (2.93)	5.02	3.08	7.35	2.15 (1.59)	1.71	0.95	2.82	2.96 (2.40)	2.25	1.17	3.90	4.13 (2.16)	3.82	2.43	5.54
32	21.05 (11.15)	19.21	12.25	28.31	1.12 (0.57)	1.05	0.67	1.48	5.48 (2.93)	5.04	3.11	7.35	2.15 (1.59)	1.71	0.95	2.82	2.96 (2.39)	2.26	1.18	3.89	4.14 (2.16)	3.82	2.44	5.54

33	21.06 (11.15)	19.29	12.27	28.31	1.12 (0.57)	1.05	0.67	1.48	5.49 (2.93)	5.05	3.11	7.34	2.15 (1.58)	1.72	0.95	2.82	2.96 (2.39)	2.26	1.19	3.90	4.14 (2.16)	3.82	2.44	5.54
34	21.12 (11.19)	19.42	12.25	28.43	1.12 (0.57)	1.06	0.67	1.49	5.51 (2.93)	5.08	3.11	7.39	2.16 (1.59)	1.74	0.95	2.83	2.97 (2.39)	2.28	1.19	3.92	4.15 (2.17)	3.83	2.43	5.57
35	21.06 (11.20)	19.26	12.16	28.41	1.12 (0.57)	1.05	0.66	1.48	5.49 (2.94)	5.04	3.10	7.37	2.15 (1.60)	1.71	0.95	2.83	2.97 (2.40)	2.26	1.19	3.92	4.13 (2.17)	3.80	2.42	5.55
36	21.03 (11.26)	19.15	12.09	28.44	1.11 (0.57)	1.05	0.66	1.48	5.48 (2.94)	5.02	3.09	7.37	2.16 (1.61)	1.71	0.94	2.85	2.98 (2.43)	2.25	1.18	3.93	4.12 (2.18)	3.78	2.40	5.54

Abbreviations: $PM_{2.5}$: particulate matter with aerodynamic diameter less than 2.5 μm; BC: black carbon; OM: organic matter; NH_4^+: ammonium; NO_3^-: nitrate; SO_4^{2-}: sulfate; SD: standard deviation.

Table S4: Distributions of NO_2 and ambient temperature by gestational week.

Gestational week	NO_2 (μg/m^3)				Temperature (°C)			
	Mean (SD)	Median	P25	P75	Mean (SD)	Median	P25	P75
1	34.75 (14.91)	31.82	24.12	42.51	23.97 (5.90)	24.55	19.62	29.48
2	34.54 (14.64)	31.75	24.09	42.24	23.93 (5.92)	24.51	19.59	29.44
3	34.47 (14.46)	31.79	24.22	41.94	23.87 (5.92)	24.47	19.56	29.39
4	34.54 (14.41)	31.90	24.33	41.97	23.82 (5.92)	24.44	19.51	29.33
5	34.55 (14.37)	31.96	24.33	42.01	23.82 (5.92)	24.42	19.52	29.33
6	34.46 (14.22)	31.96	24.33	41.83	23.81 (5.88)	24.38	19.45	29.30
7	34.35 (13.98)	31.95	24.33	41.73	23.80 (5.86)	24.39	19.48	29.27
8	34.16 (13.98)	31.83	24.19	41.41	23.81 (5.86)	24.46	19.43	29.25
9	33.93 (13.85)	31.68	24.06	41.08	23.78 (5.88)	24.45	19.34	29.25
10	33.75 (13.72)	31.57	23.95	40.90	23.77 (5.91)	24.42	19.25	29.28
11	33.67 (13.67)	31.49	23.87	40.76	23.77 (5.90)	24.43	19.25	29.28
12	33.62 (13.57)	31.47	23.82	40.78	23.78 (5.91)	24.48	19.21	29.28
13	33.48 (13.46)	31.34	23.75	40.75	23.79 (5.92)	24.49	19.21	29.30
14	33.38 (13.50)	31.19	23.55	40.69	23.83 (5.92)	24.55	19.19	29.33
15	33.24 (13.38)	31.07	23.46	40.64	23.84 (5.93)	24.53	19.20	29.35
16	33.13 (13.32)	31.01	23.37	40.54	23.89 (5.92)	24.56	19.23	29.37
17	33.07 (13.30)	30.99	23.30	40.41	23.91 (5.92)	24.57	19.25	29.40
18	32.97 (13.23)	30.89	23.29	40.30	23.96 (5.91)	24.62	19.27	29.42
19	32.75 (12.99)	30.70	23.19	39.98	23.98 (5.92)	24.67	19.31	29.46
20	32.69 (12.94)	30.67	23.23	39.83	24.03 (5.91)	24.73	19.34	29.47
21	32.61 (12.93)	30.58	23.21	39.58	24.08 (5.90)	24.80	19.43	29.52

22	32.50 (12.88)	30.54	23.12	39.41	24.12 (5.91)	24.87	19.50	29.56
23	32.42 (12.73)	30.42	23.17	39.28	24.16 (5.90)	24.92	19.59	29.59
24	32.40 (12.66)	30.42	23.22	39.24	24.22 (5.87)	24.98	19.68	29.61
25	32.30 (12.49)	30.33	23.26	38.98	24.27 (5.86)	25.04	19.82	29.62
26	32.30 (12.51)	30.30	23.28	38.93	24.35 (5.83)	25.17	19.98	29.63
27	32.17 (12.34)	30.25	23.25	38.72	24.39 (5.82)	25.19	20.04	29.66
28	32.14 (12.31)	30.19	23.23	38.65	24.43 (5.81)	25.26	20.10	29.67
29	32.06 (12.24)	30.10	23.21	38.56	24.47 (5.76)	25.31	20.21	29.65
30	32.03 (12.25)	30.10	23.23	38.46	24.51 (5.75)	25.32	20.29	29.68
31	32.00 (12.31)	30.05	23.12	38.47	24.53 (5.76)	25.37	20.31	29.73
32	31.94 (12.30)	29.99	23.04	38.42	24.56 (5.75)	25.36	20.36	29.76
33	31.95 (12.38)	29.98	22.98	38.53	24.57 (5.75)	25.35	20.34	29.77
34	31.98 (12.41)	30.09	22.93	38.71	24.60 (5.73)	25.37	20.38	29.77
35	31.94 (12.47)	29.98	22.87	38.68	24.59 (5.74)	25.35	20.31	29.77
36	31.92 (12.61)	29.86	22.66	38.79	24.57 (5.76)	25.32	20.29	29.78

Abbreviations: NO_2: nitrogen dioxide; SD: standard deviation.

Table S5: Correlation coefficients between air pollutants and ambient temperature during pregnancy.

	$PM_{2.5}$	BC	OM	NH_4^+	NO_3^-	SO_4^{2-}	NO_2	Temperature
$PM_{2.5}$	1.000	-	-	-	-	-	-	-
BC	0.959	1.000	-	-	-	-	-	-
OM	0.975	0.992	1.000	-	-	-	-	-
NH_4^+	0.827	0.712	0.764	1.000	-	-	-	-
NO_3^-	0.849	0.721	0.775	0.990	1.000	-	-	-
SO_4^{2-}	0.970	0.959	0.968	0.843	0.848	1.000	-	-
NO_2	0.635	0.598	0.582	0.423	0.509	0.574	1.000	-
Temperature	-0.616	-0.458	-0.513	-0.829	-0.852	-0.642	-0.431	1.000

Abbreviations: $PM_{2.5}$: particulate matter with aerodynamic diameter less than 2.5 µm; BC: black carbon; OM: organic matter; NH_4^+: ammonium; NO_3^-: nitrate; SO_4^{2-}: sulfate; NO_2: nitrogen dioxide.

Table S6: Cumulative risk of birth at gestational weeks 28-36 under natural course and 10% reduction intervention.

Gestational week	Natural course	$PM_{2.5}$ and its constituents					
		$PM_{2.5}$	BC	OM	NH_4^+	NO_3^-	SO_4^{2-}
28	0.00053	0.00046	0.00059	0.00057	0.00049	0.00049	0.00058
29	0.00127	0.00139	0.00151	0.00148	0.00135	0.00140	0.00150
30	0.00220	0.00241	0.00252	0.00246	0.00232	0.00242	0.00251
31	0.00391	0.00407	0.00425	0.00417	0.00405	0.00408	0.00432
32	0.00625	0.00603	0.00637	0.00631	0.00616	0.00612	0.00648
33	0.01016	0.00996	0.01013	0.00998	0.00985	0.00976	0.01014
34	0.01850	0.01780	0.01769	0.01754	0.01810	0.01796	0.01792
35	0.03270	0.03307	0.03250	0.03245	0.03280	0.03254	0.03273
36	0.06508	0.06371	0.06446	0.06409	0.06387	0.06380	0.06452

Abbreviations: $PM_{2.5}$: particulate matter with aerodynamic diameter less than 2.5 μm; BC: black carbon; OM: organic matter; NH_4^+: ammonium; NO_3^-: nitrate; SO_4^{2-}: sulfate.

Table S7: Cumulative risk of birth at gestational weeks 28-36 under natural course and 20% reduction intervention.

Gestational week	Natural course	$PM_{2.5}$ and its constituents					
		$PM_{2.5}$	BC	OM	NH_4^+	NO_3^-	SO_4^{2-}
28	0.00053	0.00039	0.00064	0.00062	0.00045	0.00044	0.00063
29	0.00127	0.00152	0.00176	0.00170	0.00144	0.00154	0.00174
30	0.00220	0.00263	0.00285	0.00273	0.00244	0.00264	0.00282
31	0.00391	0.00424	0.00459	0.00444	0.00420	0.00426	0.00473
32	0.00625	0.00583	0.00651	0.00638	0.00607	0.00601	0.00672
33	0.01016	0.00978	0.01011	0.00982	0.00955	0.00937	0.01013
34	0.01850	0.01716	0.01690	0.01662	0.01771	0.01743	0.01736
35	0.03270	0.03350	0.03233	0.03223	0.03291	0.03240	0.03279
36	0.06508	0.06241	0.06387	0.06315	0.06268	0.06254	0.06398

Abbreviations: $PM_{2.5}$: particulate matter with aerodynamic diameter less than 2.5 μm; BC: black carbon; OM: organic matter; NH_4^+: ammonium; NO_3^-: nitrate; SO_4^{2-}: sulfate.

Table S8: Cumulative risk of birth at gestational weeks 28-36 under natural course and 40% reduction intervention.

Gestational week	Natural course	$PM_{2.5}$ and its constituents					
		$PM_{2.5}$	BC	OM	NH_4^+	NO_3^-	SO_4^{2-}
28	0.00053	0.00026	0.00076	0.00071	0.00037	0.00036	0.00074
29	0.00127	0.00179	0.00227	0.00214	0.00161	0.00182	0.00222
30	0.00220	0.00310	0.00354	0.00328	0.00270	0.00311	0.00346
31	0.00391	0.00465	0.00531	0.00500	0.00451	0.00465	0.00558
32	0.00625	0.00551	0.00682	0.00658	0.00592	0.00581	0.00724
33	0.01016	0.00951	0.01014	0.00956	0.00897	0.00864	0.01019
34	0.01850	0.01599	0.01541	0.01488	0.01697	0.01644	0.01630
35	0.03270	0.03450	0.03207	0.03191	0.03318	0.03219	0.03296
36	0.06508	0.06000	0.06279	0.06138	0.06034	0.06008	0.06297

Abbreviations: $PM_{2.5}$: particulate matter with aerodynamic diameter less than 2.5 μm; BC: black carbon; OM: organic matter; NH_4^+: ammonium; NO_3^-: nitrate; SO_4^{2-}: sulfate.

Table S9: Cumulative risk of birth at gestational weeks 28-36 under natural course and 50% reduction intervention.

Gestational week	Natural course	$PM_{2.5}$ and its constituents					
		$PM_{2.5}$	BC	OM	NH_4^+	NO_3^-	SO_4^{2-}
28	0.00053	0.00020	0.00081	0.00075	0.00034	0.00032	0.00080
29	0.00127	0.00193	0.00253	0.00237	0.00170	0.00197	0.00247
30	0.00220	0.00335	0.00389	0.00357	0.00283	0.00335	0.00379
31	0.00391	0.00488	0.00568	0.00531	0.00468	0.00486	0.00602
32	0.00625	0.00539	0.00700	0.00671	0.00586	0.00573	0.00753
33	0.01016	0.00941	0.01018	0.00946	0.00869	0.00830	0.01024
34	0.01850	0.01547	0.01471	0.01405	0.01662	0.01597	0.01581
35	0.03270	0.03507	0.03199	0.03180	0.03334	0.03210	0.03308
36	0.06508	0.05889	0.06230	0.06056	0.05920	0.05889	0.06249

Abbreviations: $PM_{2.5}$: particulate matter with aerodynamic diameter less than 2.5 μm; BC: black carbon; OM: organic matter; NH_4^+: ammonium; NO_3^-: nitrate; SO_4^{2-}: sulfate.

Table S10: Cumulative risk of birth at gestational weeks 28-36 under natural course and 60% reduction intervention.

Gestational week	Natural course	$PM_{2.5}$ and its constituents					
		$PM_{2.5}$	BC	OM	NH_4^+	NO_3^-	SO_4^{2-}
28	0.00053	0.00014	0.00087	0.00080	0.00030	0.00028	0.00085
29	0.00127	0.00209	0.00279	0.00260	0.00180	0.00212	0.00272
30	0.00220	0.00360	0.00425	0.00386	0.00297	0.00360	0.00413
31	0.00391	0.00512	0.00607	0.00562	0.00484	0.00507	0.00646
32	0.00625	0.00529	0.00720	0.00685	0.00581	0.00566	0.00782
33	0.01016	0.00934	0.01024	0.00938	0.00843	0.00796	0.01031
34	0.01850	0.01500	0.01403	0.01327	0.01628	0.01551	0.01533
35	0.03270	0.03569	0.03193	0.03172	0.03351	0.03204	0.03322
36	0.06508	0.05784	0.06185	0.05978	0.05807	0.05771	0.06204

Abbreviations: $PM_{2.5}$: particulate matter with aerodynamic diameter less than 2.5 μm; BC: black carbon; OM: organic matter; NH_4^+: ammonium; NO_3^-: nitrate; SO_4^{2-}: sulfate.

Table S11: Cumulative risk of birth at gestational weeks 28-36 under natural course and 80% reduction intervention.

Gestational week	Natural course	PM2.5 and its constituents					
		$PM_{2.5}$	BC	OM	NH_4^+	NO_3^-	SO_4^{2-}
28	0.00053	0.00002	0.00099	0.00090	0.00022	0.00020	0.00097
29	0.00127	0.00241	0.00333	0.00308	0.00199	0.00244	0.00322
30	0.00220	0.00415	0.00500	0.00446	0.00325	0.00412	0.00481
31	0.00391	0.00567	0.00687	0.00628	0.00519	0.00553	0.00738
32	0.00625	0.00517	0.00764	0.00720	0.00572	0.00556	0.00846
33	0.01016	0.00928	0.01041	0.00927	0.00792	0.00734	0.01052
34	0.01850	0.01419	0.01276	0.01180	0.01564	0.01465	0.01444
35	0.03270	0.03709	0.03190	0.03168	0.03390	0.03197	0.03357
36	0.06508	0.05594	0.06104	0.05835	0.05587	0.05542	0.06121

Abbreviations: $PM_{2.5}$: particulate matter with aerodynamic diameter less than 2.5 μm; BC: black carbon; OM: organic matter; NH_4^+: ammonium; NO_3^-: nitrate; SO_4^{2-}: sulfate.

Table S12: Cumulative risk of birth at gestational weeks 28-36 under natural course and 90% reduction intervention.

Gestational week	Natural course	$PM_{2.5}$ and its constituents					
		$PM_{2.5}$	BC	OM	NH_4^+	NO_3^-	SO_4^{2-}
28	0.00053	-0.00003	0.00106	0.00095	0.00019	0.00016	0.00102
29	0.00127	0.00259	0.00361	0.00333	0.00209	0.00261	0.00348
30	0.00220	0.00444	0.00538	0.00477	0.00339	0.00439	0.00516
31	0.00391	0.00598	0.00729	0.00662	0.00537	0.00577	0.00785
32	0.00625	0.00515	0.00789	0.00740	0.00569	0.00553	0.00880
33	0.01016	0.00929	0.01052	0.00925	0.00768	0.00705	0.01065
34	0.01850	0.01385	0.01216	0.01111	0.01534	0.01425	0.01403
35	0.03270	0.03786	0.03193	0.03171	0.03411	0.03196	0.03378
36	0.06508	0.05509	0.06069	0.05770	0.05478	0.05426	0.06083

Abbreviations: $PM_{2.5}$: particulate matter with aerodynamic diameter less than 2.5 μm; BC: black carbon; OM: organic matter; NH_4^+: ammonium; NO_3^-: nitrate; SO_4^{2-}: sulfate.

Table S13: Cumulative risk difference and 95% CI per thousand births at gestational week 36 under 40% reduction in $PM_{2.5}$ and its constituents' intervention in stratified analysis.

Stratifying factors	Subgroup	RD (95% CI)	*P*-interaction
**Infant sex**			
$PM_{2.5}$	Male	-1.63 (-19.90, 18.02)	0.63
	Female	-7.86 (-24.31, 10.17)	
BC	Male	-0.37 (-16.86, 20.07)	0.76
	Female	-3.94 (-16.95, 9.53)	
OM	Male	-1.86 (-19.03, 17.10)	0.78
	Female	-5.35 (-22.96, 10.56)	
NH_4^+	Male	-5.69 (-18.68, 12.14)	0.77
	Female	-2.55 (-15.96, 12.63)	
NO_3^-	Male	-6.63 (-21.42, 7.98)	0.68
	Female	-1.93 (-17.11, 15.65)	
SO_4^{2-}	Male	-1.01 (-18.16, 16.11)	0.88
	Female	-2.98 (-19.38, 16.74)	
**Maternal age**			
$PM_{2.5}$	< 30 y	-3.02 (-12.43, 15.87)	0.88
	≥ 30 y	-4.95 (-23.56, 18.82)	
BC	< 30 y	-1.73 (-18.78, 16.37)	0.96
	≥ 30 y	-2.30 (-18.25, 16.79)	
OM	< 30 y	-3.90 (-18.20, 13.71)	0.94
	≥ 30 y	-2.94 (-20.08, 15.42)	

NH_4^+	< 30 y	-3.61 (-17.64, 9.89)	0.91
	≥ 30 y	-4.79 (-21.20, 7.14)	
NO_3^-	< 30 y	-3.62 (-18.53, 10.09)	0.89
	≥ 30 y	-5.05 (-19.83, 7.70)	
SO_4^{2-}	< 30 y	-1.51 (-14.92, 13.84)	0.98
	≥ 30 y	-1.93 (-25.82, 18.06)	

Abbreviations: CI: confidence interval; $PM_{2.5}$: particulate matter with aerodynamic diameter less than 2.5 µm; BC: black carbon; OM: organic matter; NH_4^+: ammonium; NO_3^-: nitrate; SO_4^{2-}: sulfate.

Table S14: Cumulative risk difference and 95% CI per thousand births at gestational week 36 under 20% reduction intervention in sensitivity analyses (1), (2) and (3).

	$PM_{2.5}$ and its constituents					
	$PM_{2.5}$	BC	OM	NH_4^+	NO_3^-	SO_4^{2-}
(1)	-4.49<br>(-9.78, 3.05)	-2.03<br>(-8.50, 9.45)	-2.57<br>(-7.50, 3.91)	-4.33<br>(-9.40, -0.44)	-4.14<br>(-9.73, 0.37)	-2.72<br>(-6.66, 8.26)
(2)	-1.33<br>(-7.00, 4.07)	0.75<br>(-4.19, 7.79)	-0.13<br>(-4.77, 6.08)	-3.56<br>(-10.11, -0.70)	-4.91<br>(-11.74, -2.33)	-1.11<br>(-7.38, 5.64)
(3)	-4.13<br>(-11.26, 2.11)	-3.42<br>(-9.22, 2.19)	-3.88<br>(-10.64, 3.59)	-4.05<br>(-9.83, 0.15)	-4.07<br>(-9.16, -1.03)	-3.47<br>(-9.93, 2.20)

Abbreviations: CI: confidence interval; $PM_{2.5}$: particulate matter with aerodynamic diameter less than 2.5 µm; BC: black carbon; OM: organic matter; NH_4^+: ammonium; NO_3^-: nitrate; SO_4^{2-}: sulfate.

Cumulative risk differences were calculated by comparing each intervention strategies with no intervention (natural course).

(1) Excluding infants with low birth weight (< 2500 g) or macrosomia (≥ 4000 g);

(2) Excluding nitrogen dioxide from the models;

(3) Redefinition of preterm birth.

Table S15: Cumulative risk of birth at gestational weeks 28-36 under natural course and 20% reduction intervention in sensitivity analysis excluding infants with low birth weight or macrosomia.

Gestational week	Natural course	PM$_{2.5}$ and its constituents					
		PM$_{2.5}$	BC	OM	NH_4^+	NO_3^-	SO_4^{2-}
28	0.00004	0.00010	0.00013	0.00020	0.00003	0.00001	0.00012
29	0.00003	0.00010	0.00012	0.00019	0.00003	0.00001	0.00011
30	0.00006	0.00019	0.00016	0.00022	0.00006	0.00002	0.00026
31	0.00012	0.00018	0.00016	0.00022	0.00014	0.00008	0.00029
32	0.00022	0.00020	0.00030	0.00034	0.00014	0.00006	0.00041
33	0.00062	0.00038	0.00047	0.00053	0.00037	0.00026	0.00054
34	0.00232	0.00146	0.00227	0.00233	0.00178	0.00163	0.00244
35	0.00885	0.00846	0.00884	0.00902	0.00791	0.00771	0.00860
36	0.03327	0.02878	0.03124	0.03070	0.02894	0.02913	0.03055

Abbreviations: PM$_{2.5}$: particulate matter with aerodynamic diameter less than 2.5 μm; BC: black carbon; OM: organic matter; NH_4^+: ammonium; NO_3^-: nitrate; SO_4^{2-}: sulfate.

Table S16: Cumulative risk of birth at gestational weeks 28-36 under natural course and 20% reduction in sensitivity analysis excluding nitrogen dioxide in the models.

Gestational week	Natural course	$PM_{2.5}$ and its constituents					
		$PM_{2.5}$	BC	OM	NH_4^+	NO_3^-	SO_4^{2-}
28	0.00053	0.00036	0.00061	0.00059	0.00044	0.00043	0.00061
29	0.00127	0.00144	0.00166	0.00162	0.00147	0.00154	0.00169
30	0.00220	0.00259	0.00277	0.00268	0.00254	0.00267	0.00283
31	0.00391	0.00417	0.00460	0.00441	0.00419	0.00419	0.00482
32	0.00625	0.00595	0.00667	0.00658	0.00615	0.00604	0.00691
33	0.01016	0.00990	0.01063	0.01035	0.00949	0.00911	0.01056
34	0.01850	0.01758	0.01823	0.01781	0.01730	0.01686	0.01841
35	0.03270	0.03418	0.03414	0.03382	0.03194	0.03130	0.03385
36	0.06508	0.06375	0.06583	0.06495	0.06152	0.06017	0.06397

Abbreviations: $PM_{2.5}$: particulate matter with aerodynamic diameter less than 2.5 μm; BC: black carbon; OM: organic matter; NH_4^+: ammonium; NO_3^-: nitrate; SO_4^{2-}: sulfate.

Table S17: Distribution of gestational weeks in sensitivity analysis redefining preterm birth using a 20-week threshold.

Gestational week	Number of deliveries (%)
24	22 (0.02%)
25	40 (0.03%)
26	52 (0.04%)
27	69 (0.05%)
28	78 (0.05%)
29	110 (0.07%)
30	138 (0.09%)
31	254 (0.17%)
32	348 (0.23%)
33	584 (0.39%)
34	1,248 (0.84%)
35	2,133 (1.44%)
36	4,864 (3.28%)
⩾ 37	138,311 (93.30%)

Table S18: Cumulative risk of birth at gestational weeks 24-36 under natural course and 20% reduction in sensitivity analysis using a 20-week definition of preterm birth.

Gestational week	Natural course	PM$_{2.5}$ and its constituents					
		PM$_{2.5}$	BC	OM	NH_4^+	NO_3^-	SO_4^{2-}
24	0.00015	-0.00008	-0.00013	0.00005	-0.00011	0.00008	-0.00012
25	0.00042	-0.00026	-0.00037	-0.00004	-0.00031	-0.00001	-0.00045
26	0.00077	-0.00083	-0.00063	0.00004	-0.00049	0.00003	-0.00073
27	0.00129	-0.00096	-0.00106	-0.00028	-0.00096	-0.00035	-0.00122
28	0.00181	-0.00063	-0.00042	0.00017	-0.00035	0.00010	-0.00059
29	0.00256	0.00084	0.00070	0.00115	0.00073	0.00119	0.00052
30	0.00349	0.00164	0.00179	0.00216	0.00177	0.00229	0.00160
31	0.00520	0.00341	0.00353	0.00392	0.00348	0.00392	0.00351
32	0.00753	0.00549	0.00545	0.00579	0.00542	0.00566	0.00550
33	0.01144	0.00875	0.00907	0.00927	0.00887	0.00904	0.00892
34	0.01976	0.01569	0.01586	0.01742	0.01572	0.01715	0.01625
35	0.03395	0.03117	0.03130	0.03263	0.03134	0.03213	0.03163
36	0.06629	0.06216	0.06287	0.06241	0.06224	0.06222	0.06282

Abbreviations: PM$_{2.5}$: particulate matter with aerodynamic diameter less than 2.5 µm; BC: black carbon; OM: organic matter; NH_4^+: ammonium; NO_3^-: nitrate; SO_4^{2-}: sulfate.

Table S19: Cumulative risk difference and 95% CI per thousand births at gestational week 36 under 20% reduction intervention in two-constituent models.

	BC	OM	NH_4^+	NO_3^-	SO_4^{2-}
+ BC	-	-56.40<br>(-117.42, -50.59)	-4.88<br>(-12.24, -0.19)	-6.32<br>(-15.57, -2.25)	-1.26<br>(-21.27, 26.86)
+ OM	61.08<br>(55.93, 140.56)	-	-3.77<br>(-12.25, 0.64)	-5.13<br>(-15.38, 1.12)	12.82<br>(0.77, 44.41)
+ NH_4^+	3.13<br>(-2.71, 11.60)	1.05<br>(-6.81, 10.44)	-	-3.56<br>(-31.66, 16.50)	4.48<br>(-1.98, 15.34)
+ NO_3^-	4.24<br>(-0.59, 12.75)	2.19<br>(-4.19, 9.52)	3.30<br>(-7.76, 33.20)	-	5.23<br>(-4.45, 18.55)
+ SO_4^{2-}	1.02<br>(-18.74, 24.65)	-11.27<br>(-36.06, 1.48)	-4.79<br>(-12.10, 2.14)	-5.41<br>(-15.80, 0.26)	-

Abbreviations: CI: confidence interval; BC: black carbon; OM: organic matter; NH_4^+: ammonium; NO_3^-: nitrate; SO_4^{2-}: sulfate.

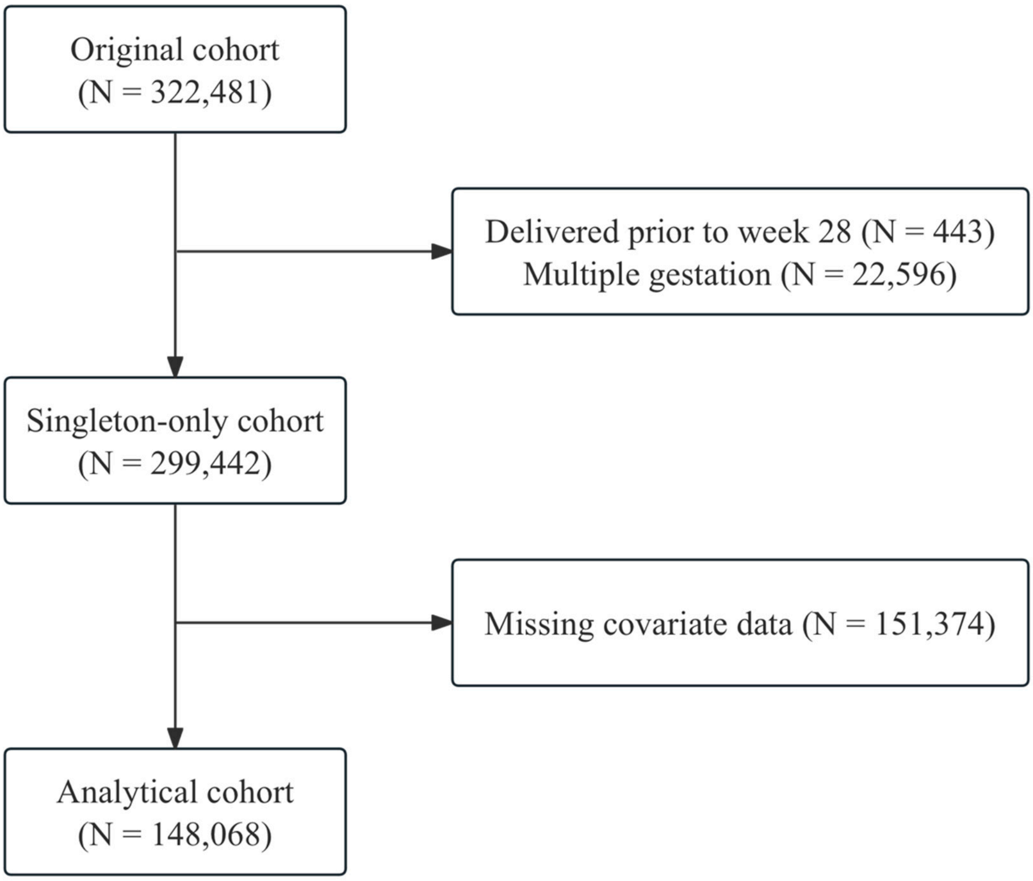

Figure S1: The process of participant selection.

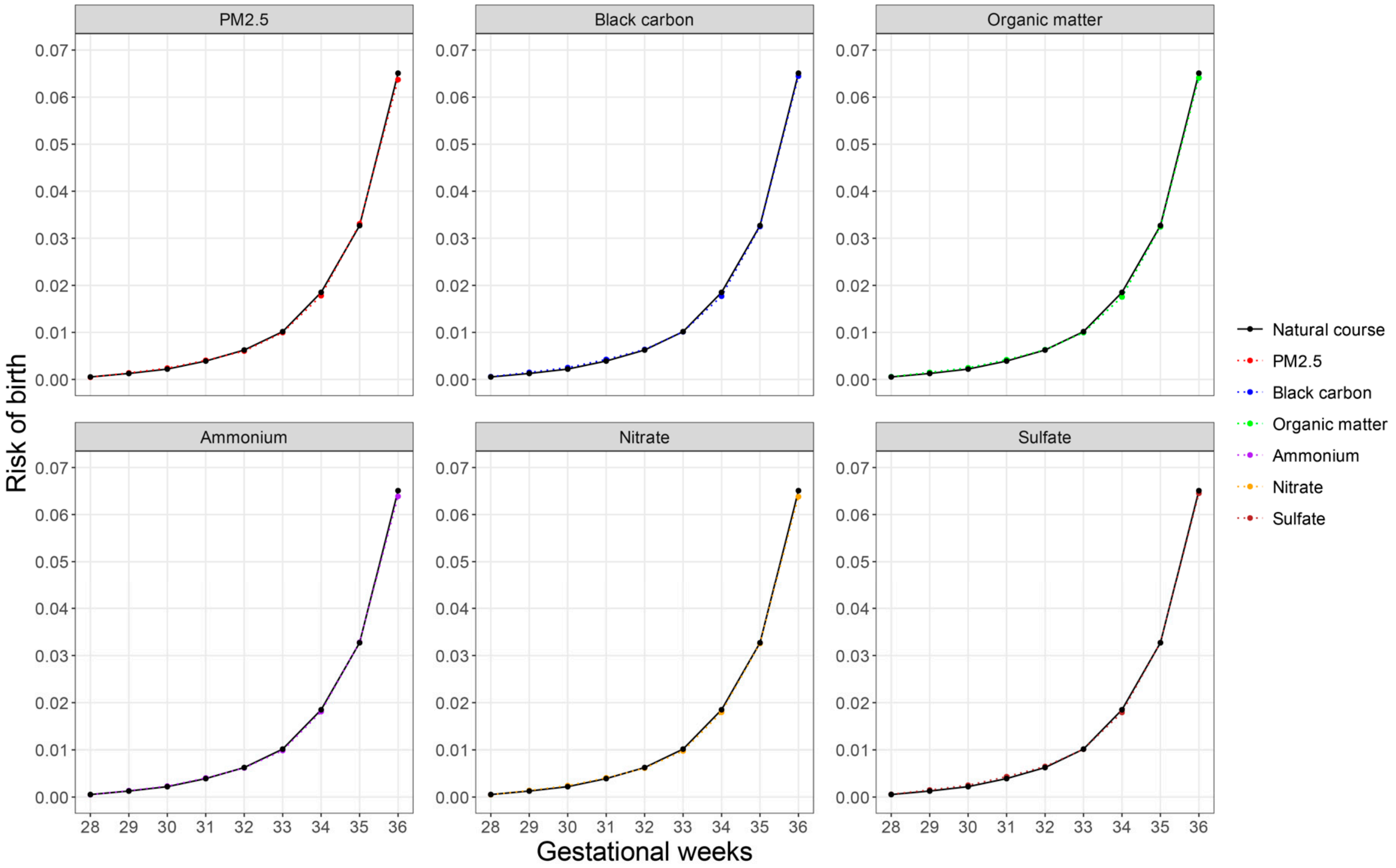

Figure S2: Cumulative risk of birth at gestational weeks 28-36 under natural course and 10% reduction intervention.

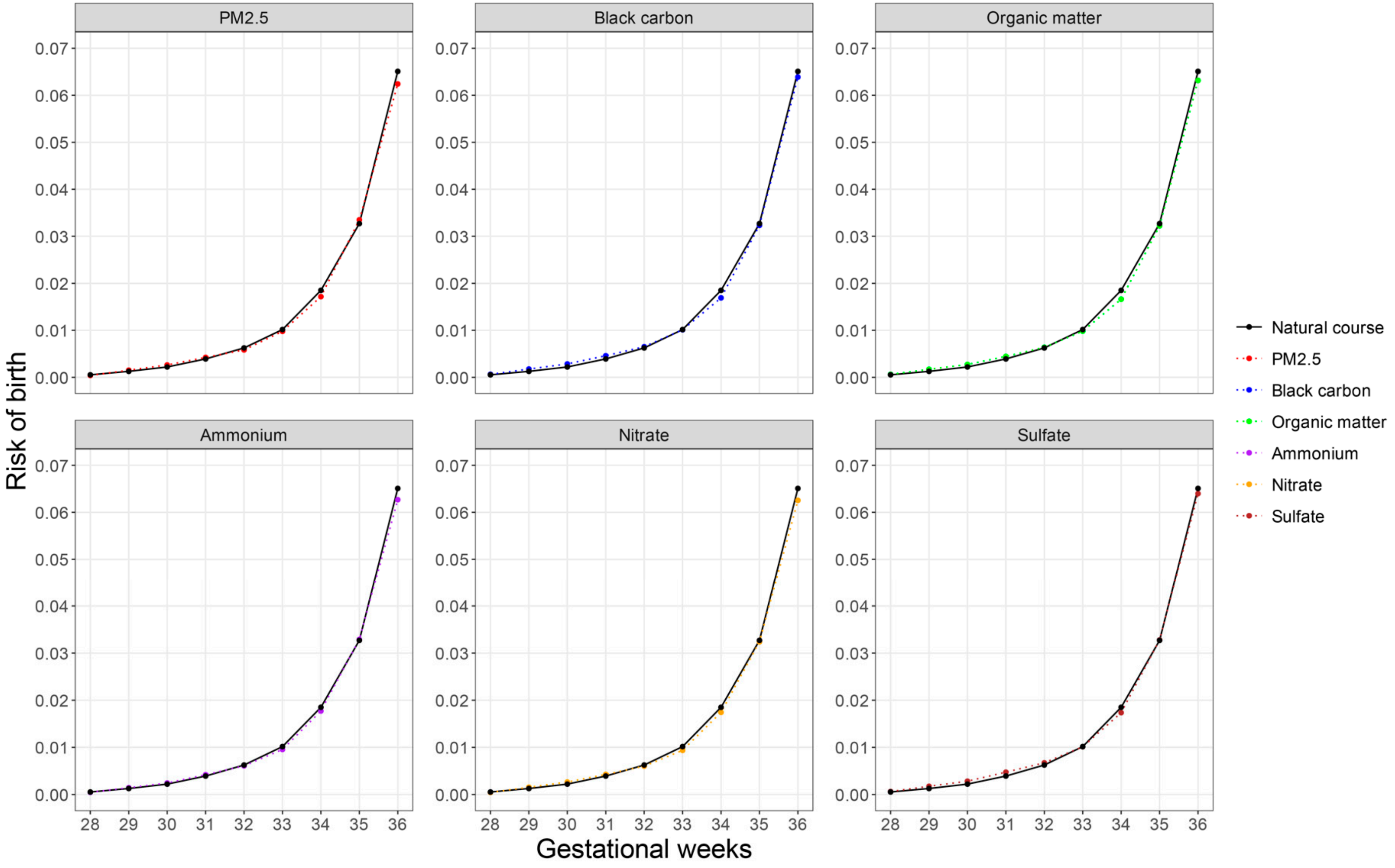

Figure S3: Cumulative risk of birth at gestational weeks 28-36 under natural course and 20% reduction intervention.

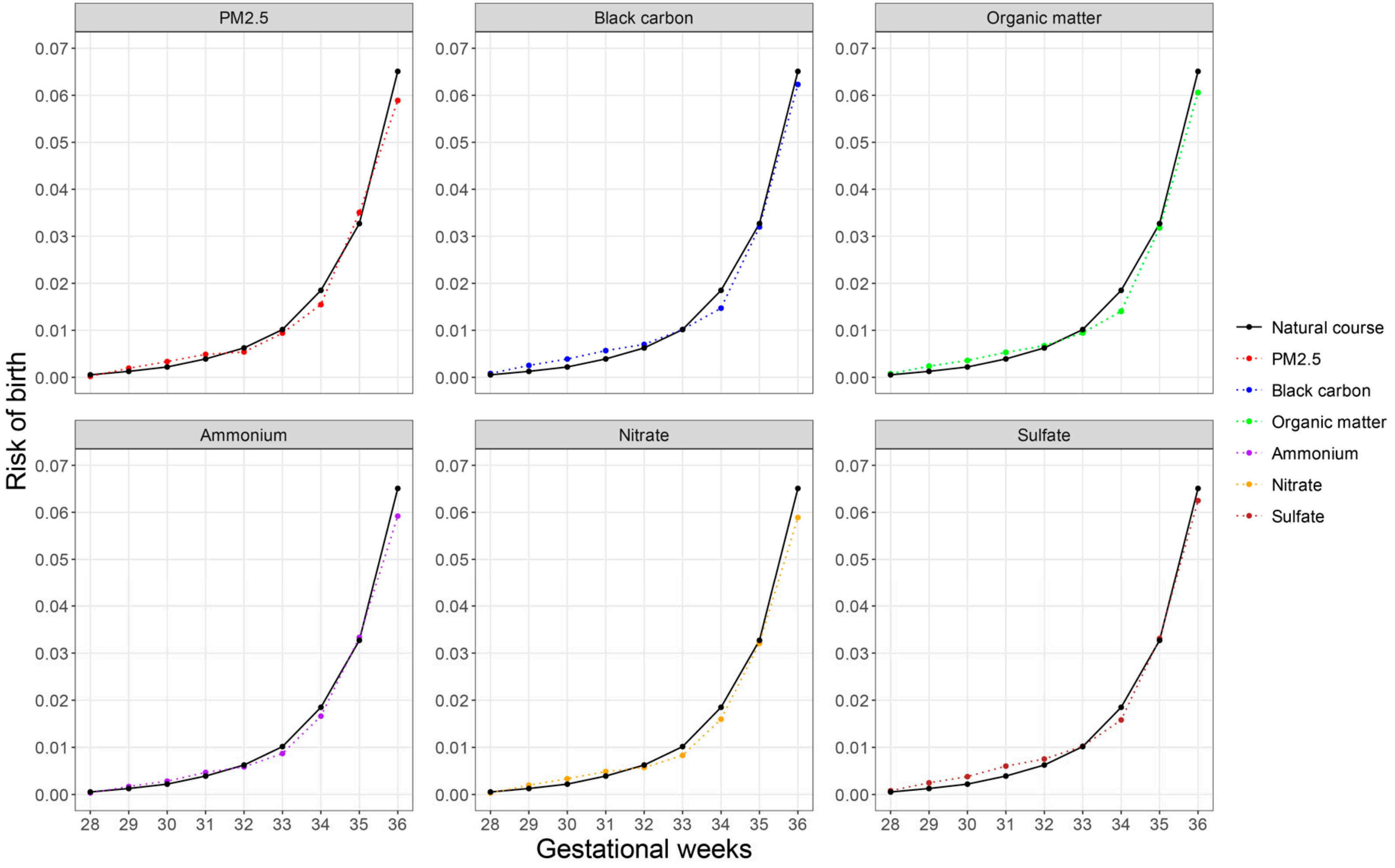

Figure S4: Cumulative risk of birth at gestational weeks 28-36 under natural course and 50% reduction intervention.

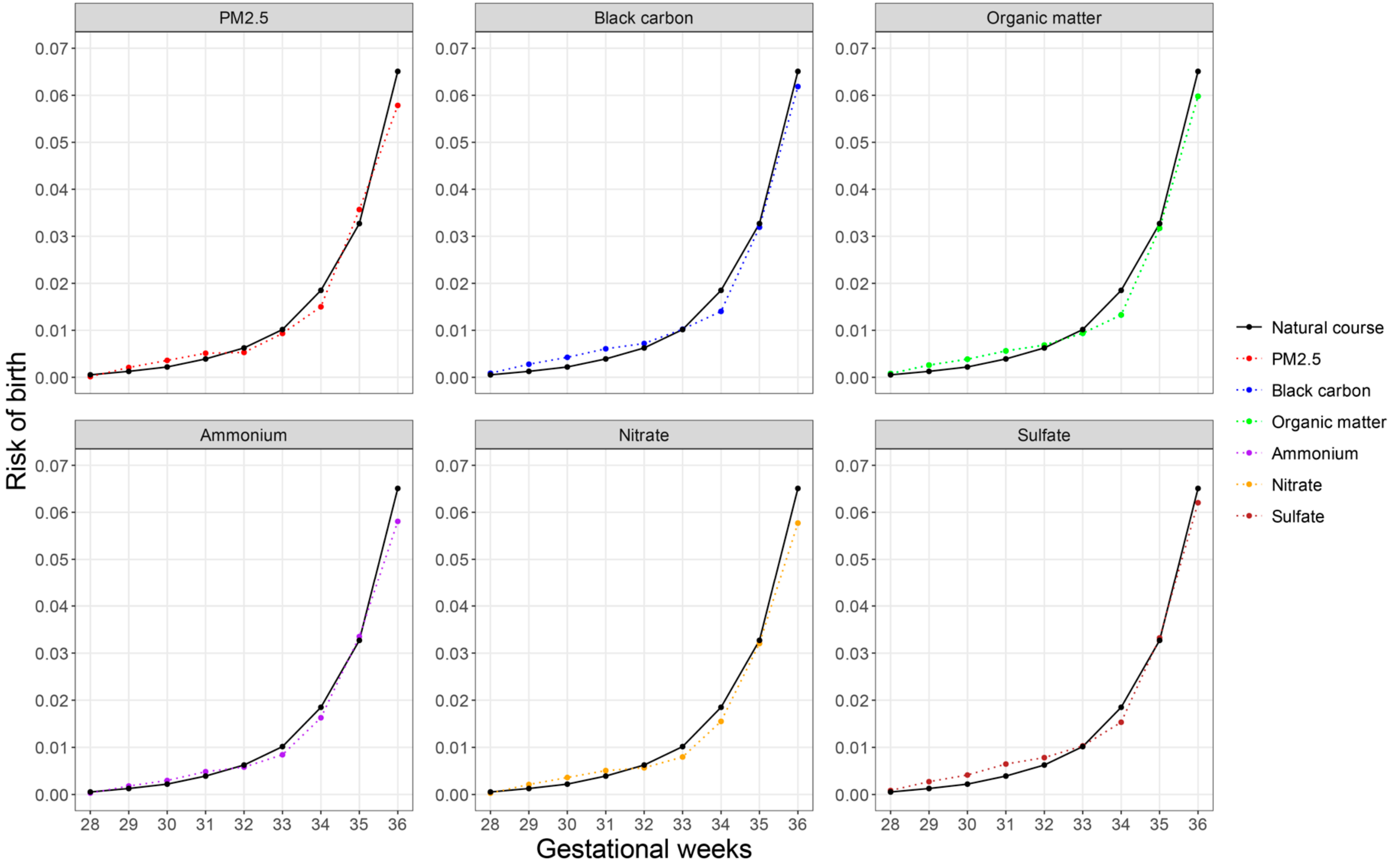

Figure S5: Cumulative risk of birth at gestational weeks 28-36 under natural course and 60% reduction intervention.

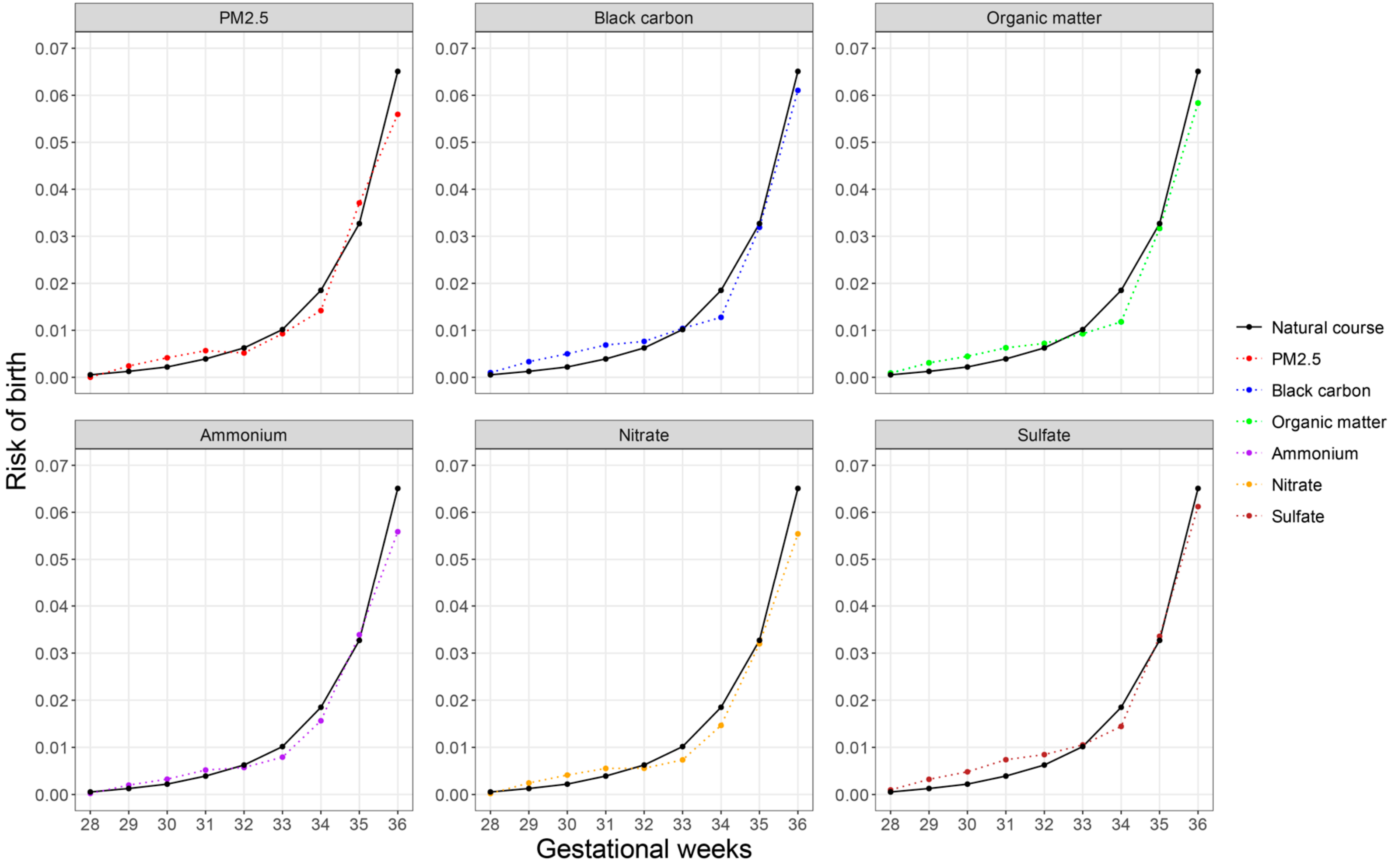

Figure S6: Cumulative risk of birth at gestational weeks 28-36 under natural course and 80% reduction intervention.

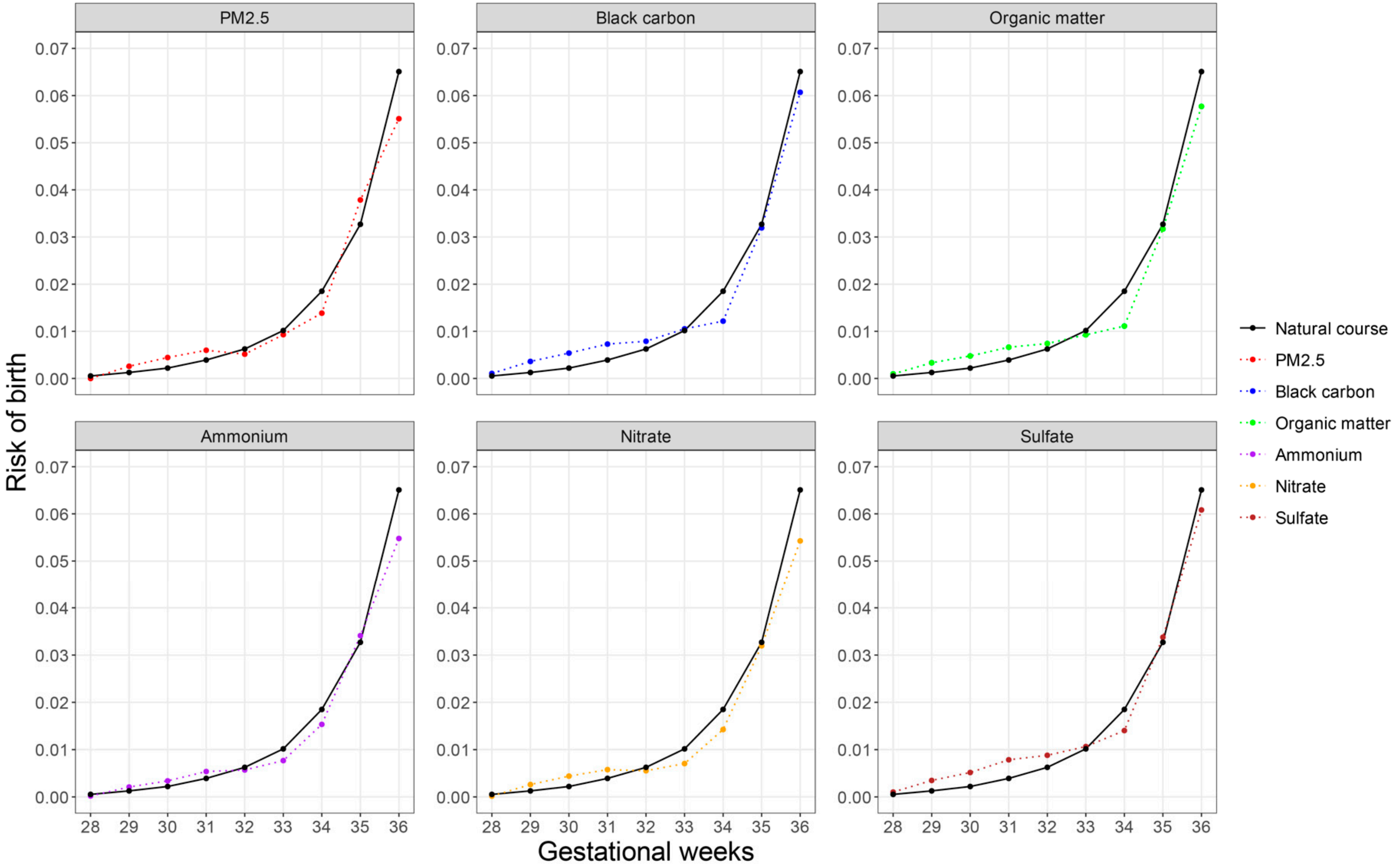

Figure S7: Cumulative risk of birth at gestational weeks 28-36 under natural course and 90% reduction intervention.
